# Leptin signaling in the dorsomedial hypothalamus couples breathing and metabolism in obesity

**DOI:** 10.1016/j.celrep.2023.113512

**Published:** 2023-11-30

**Authors:** Mateus R. Amorim, Xin Wang, O. Aung, Shannon Bevans-Fonti, Frederick Anokye-Danso, Caitlin Ribeiro, Joan Escobar, Carla Freire, Huy Pho, Olga Dergacheva, Luiz G.S. Branco, Rexford S. Ahima, David Mendelowitz, Vsevolod Y. Polotsky

**Affiliations:** 1 Department of Medicine, Johns Hopkins University, Baltimore, MD 21224, USA; 2 Department of Anesthesiology and Critical Care Medicine, George Washington University, Washington, DC 20037, USA; 3 Department of Pharmacology and Physiology, George Washington University, Washington, DC 20037, USA; 4 University of São Paulo, Ribeirão Preto, São Paulo 14040-904, Brazil; 5 Lead contact

## Abstract

Mismatch between CO_2_ production (VCO_2_) and respiration underlies the pathogenesis of obesity hypoventilation. Leptin-mediated CNS pathways stimulate both metabolism and breathing, but interactions between these functions remain elusive. We hypothesized that LEPR^b^+ neurons of the dorsomedial hypothalamus (DMH) regulate metabolism and breathing in obesity. In diet-induced obese *Lepr*^b^*Cre* mice, chemogenetic activation of LEPR^b^+ DMH neurons increases minute ventilation (VE) during sleep, the hypercapnic ventilatory response, VCO_2_, and VE/VCO_2_, indicating that breathing is stimulated out of proportion to metabolism. The effects of chemogenetic activation are abolished by a serotonin blocker. Optogenetic stimulation of the LEPR^b^+ DMH neurons evokes excitatory postsynaptic currents in downstream serotonergic neurons of the dorsal raphe (DR). Administration of retrograde AAV harboring *Cre*-dependent caspase to the DR deletes LEPR^b^+ DMH neurons and abolishes metabolic and respiratory responses to leptin. These findings indicate that LEPR^b^+ DMH neurons match breathing to metabolism through serotonergic pathways to prevent obesity-induced hypoventilation.

## In brief

Amorim et al. show that LEPR^b^+ neurons of dorsomedial hypothalamus (DMH) neurons projecting to serotonergic dorsal raphe neurons are necessary and sufficient for coupling CO_2_ production to ventilatory responses to CO_2_. Activation of the LEPR^b^+ DMH-dorsal raphe pathway is critical for preventing obesity hypoventilation and upper airway obstruction during sleep.

## Graphical abstract

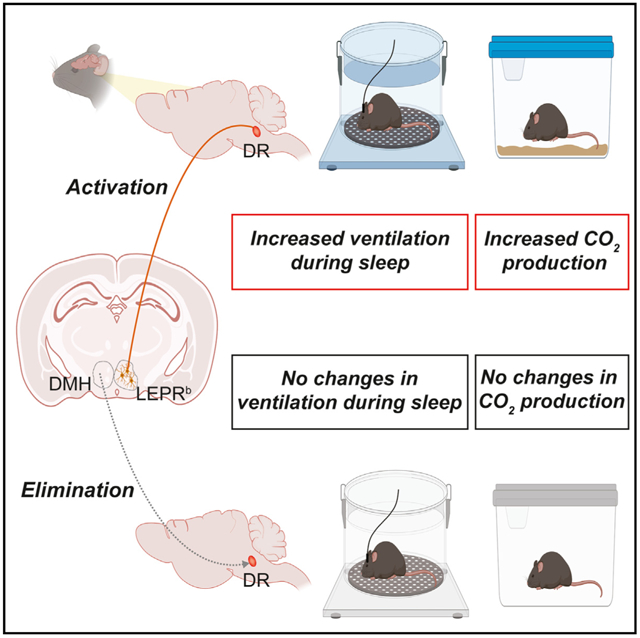

## INTRODUCTION

Obesity is a chronic disease that has reached global epidemic levels. The prevalence of obesity in the U.S. adult population is 35%–40%, leading to high morbidity and mortality.^1^ Obesity is the most common risk factor for sleep-disordered breathing (SDB), and 50% of obese individuals have SDB.^2–7^ A common type of SDB is obesity hypoventilation syndrome (OHS), defined as daytime hypercapnia (arterial carbon dioxide partial pressure [PaCO_2_] ≥ 45 mm Hg) in obese individuals in the absence of another explanation for hypoventilation.^8^ OHS is characterized by impaired ventilatory response to hypercarbia.^9^ Approximately 90% of OHS patients have obstructive sleep apnea (OSA), characterized by recurrent loss of upper airway muscle tone during sleep leading to upper airway collapse.^10,11^ The genioglossus muscle, an extrinsic tongue muscle responsible for the maintenance of pharyngeal patency during sleep, is highly sensitive to CO_2_.^12^ CO_2_ sensitivity is a potential therapeutic target in OSA and OHS.

We have previously developed a novel approach to rodent polysomnography by detecting upper airway obstruction in the breathby-breath analysis. Ventilation during obstructed or inspiratory flow-limited breathing reflects upper airway patency during sleep, whereas ventilation during non-flow-limited breathing is an integral measure of metabolism and control of breathing.^13–16^ Similarly to obese humans, diet-induced obese (DIO) mice show hypoventilation and upper airway obstruction during sleep, which lead to high PaCO_2_ during wakefulness.^13,17^ PaCO_2_ levels are a function of the balance between CO_2_ generation as a by-product of metabolism and CO_2_ elimination by the lungs. Specific neuronal and glial cells function as central respiratory chemoreceptors that control breathing by detecting small variations in pH/PaCO_2_.^18^ Although existing evidence sheds light on the relationship between CO_2_ production (VCO_2_) and control of breathing,^19–23^ the specific neural pathways linking these functions are not fully understood.

Leptin suppresses feeding and increases metabolic rate via neuronal circuits in the hypothalamus and medulla^24–27^ acting on the long isoform of leptin receptor (LEPR^b^).^28,29^ Leptin also stimulates control of breathing and improves upper airway patency during sleep in leptin-deficient *ob*/*ob* mice.^14,16,30,31^ However, obese patients and DIO mice have high circulating leptin levels^32,33^ and are resistant to the metabolic and respiratory effects of the hormone.^13,32–34^ There are several mechanisms of leptin resistance,^35–37^ including impaired leptin transport across the blood-brain barrier (BBB).^38,39^ Intranasal leptin administration allows to bypass the BBB^40^ and circumvents resistance to metabolic and respiratory effects of leptin in obesity.^34^

The effects of leptin on energy expenditure and VCO_2_ have been localized to the hypothalamus and, specifically, to the dorsomedial hypothalamus (DMH).^41–45^ In contrast, respiratory effects of leptin have not been definitively localized.^30,46–50^ The carotid body was initially thought to mediate the effects of leptin on respiration,^51^ but recent studies inDIO mice rejected this hypothesis.^52,53^Several publications suggested the role of the nucleus of the solitary tract (NTS), but the studies were performed under anesthesia,^54,55^ in the absence of obesity, and without parallel sleep and metabolic measurements.^50,56^ We have shown that chemogenetic activation of LEPR^b^+ NTS neurons neither increased ventilation nor improved upper airway patency during sleep with rigorous sleep measurements.^57^

Our laboratory reported that the respiratory effects of leptin may instead localize to the DMH, similar to the metabolic effects of the hormone. We have shown that intracerebroventricular administration of leptin did not affect breathing in *Lepr*^*b*^-deficient *db*/*db* mice, which hypoventilate during sleep and have elevated PaCO_2_, and the respiratory effects of leptin were restored upon selective expression of LEPR^b^ in the DMH.^47^ We hypothesize that LEPR^b^+ neurons in the DMH regulate both metabolism and breathing and that stimulation of these neurons relieves OHS and OSA.

**Figure 1.**
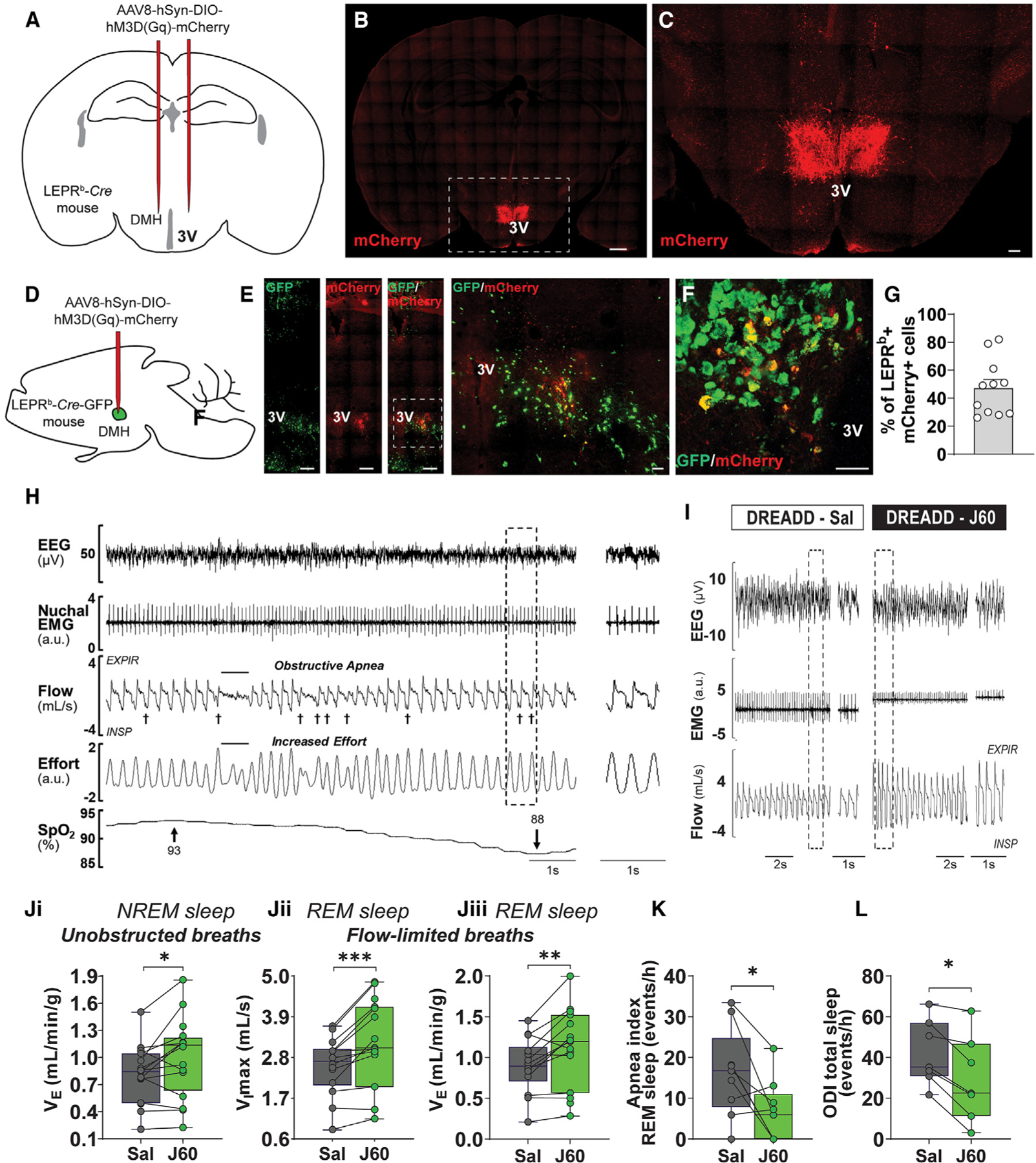
Activation of LEPR^b^ neurons in the dorsomedial hypothalamus (DMH) stimulates breathing during sleep in obese mice (A) *Cre*-dependent designer receptor exclusively activated by designer drug (DREADD; AAV8-hSyn-DIO-hM3D[Gq]-mCherry) or control virus (AAV8-hSyn-DIO-mCherry) was deployed in the DMH of *Lepr*^*b*^-*Cre* diet-induced obese (DIO) mice. 3V, third ventricle. (B) Coronal section of the mouse brain shows mCherry positivity in the DMH confirming successful DREADD transfection. Scale bar, 500 μM. (C) The outlined area is enlarged; scale bar, 100 μM. (D) Schematics of the mouse brain illustrating a site of injection of the DREADD or control virus in *Lepr*^*b*^-*Cre*-GFP DIO mouse. (E) LEPR^b^-positive neurons of the DMH (green) were transfected with DREADD (red). The orange color results from merging the red and green colors demonstrating DREADD expression in the LEPR^b^-positive neurons. Scale bars, 300 μM. The outlined area is enlarged in the right panel (scale bar, 70 μM). (F) Representative high-resolution confocal image showing DREADD expression (red) in the LEPR^b^-positive neurons (green) resulting in orange color. Scale bar, 50 μM. (G) Percentage of LEPR^b^+ mCherry+ cells (n = 6 mice). The values are expressed as means ± SE. (H) A representative screen of REM sleep in an obese *Lepr*^*b*^-*Cre* mouse with obstructive apnea. yInspiratory flow limitation. SpO_2_, oxyhemoglobin saturation. (I) Representative traces of REM sleep recordings in the same mouse expressing DREADD in the DMH after treatment with saline versus J60. (J) Minute ventilation (VE) during NREM sleep (left) and REM sleep (center) and maximal inspiratory flow (V_I_max) (right) in DIO *Lepr*^*b*^-*Cre* mice transfected with DREADD (n = 16 or 17) virus in DMH after saline and J60. (K) Number of apneas per hour (apnea index) in REM sleep. (L) Number of oxyhemoglobin desaturations ≥4% from baseline (oxygen desaturation index [ODI]) during sleep. Data are plotted using boxplots (median ± 1.5*interquartile range). *p ≤ 0.05, **p < 0.01, and ***p < 0.001 using Wilcoxon matched-pairs signed rank test.

We tested our hypothesis in a series of *in vivo* and *in vitro* studies. We measured breathing across sleep/wake states, the hypercapnic ventilatory response (HCVR), VCO_2_, and O_2_ consumption (VO_2_) in DIO *Lepr*^*b*^-*Cre* mice expressing designer receptors exclusively activated by designer drugs (DREADDs) in LEPR^b^+ neurons of the DMH (Figure 1A). Optogenetic studies in brain slices revealed numerous projections from DMH LEPR^b^+ neurons to serotonergic (5-hydroxytryptophan [5-HT+]) neurons of the dorsal raphe (DR). On the basis of the *in vitro* data, we examined if the effect of LEPR^b^+ DMH neurons on breathing can be abolished by the serotonergic blockade. The role of the novel DMH LEPR^b^+-DR pathway in matching breathing and metabolism was further tested in “loss of function” experiments in DIO *Lepr*^*b*^-*Cre* mice, in which a retrograde adeno-associated virus (AAV) harboring *Cre*-dependent caspase was administered into the DR nucleus. The relevance of chemogenetic data has been examined in experiments with intranasal leptin.

## RESULTS

### Activation of LEPR^b^+ neurons in the DMH relieved obesity hypoventilation and upper airway obstruction in obese mice

We examined the role of LEPR^b^+ neurons in the DMH in the control of breathing and upper airway function during sleep in our *Lepr*^*b*^-*Cre-GFP* DIO mouse model of OHS and OSA^13^ (Figure S1). The effect of chemogenetic activation of DMH LEPR^b^+ neurons was determined in AAV-DREADD vs. control AAV-transfected mice (Figure 1A). LEPR^b^+ neurons were abundant in the DMH (Figures 1B and 1C). Four weeks after AAV8-hSyn-DIO-hM3D(Gq)-mCherry administration, confocal images revealed co-localization (orange) of mCherry (red) and GFP (green) (Figures 1D–1F), confirming that DREADDs were successfully and selectively expressed in LEPR^b^+ DMH neurons. DREADD expression was detected in 47% ± 6% of DMH LEPR^b^+ neurons (Figure 1G). DREADDs were identified in neurons by co-localizing mCherry and NeuN (Figure S2). DREADDs were absent in astrocytes and microglia, as shown by the lack of co-localization of mCherry with GFAP and IBA-1 (Figure S2). *Lepr*^*b*^-*Cre*-*GFP* DIO mice had similar body weight, food intake, and body temperature in all treatment groups (Figure S3). Flow-limited breaths were uncommon during non-rapid-eye-movement (NREM) sleep and, therefore, were not quantified in the present study. In contrast, inspiratory flow limitation was prevalent in rapid-eye-movement (REM) sleep (Table S1). Representative polysomnography recordings of REM sleep are shown in Figure 1 H and I. In untreated obese mice, we observed apneas with typical characteristics of obstructive events in REM sleep, i.e., cessation of breathing, despite continuous respiratory effort, which led to oxyhemoglobin desaturations (Figure 1). Chemogenetic activation of LEPR^b^+ neurons in the DMH with the DREADD agonist J60 increased inspiratory flow and minute ventilation (V_E_) (Figures 1I and 1J). J60 significantly increased V_E_ during NREM sleep (Figure 1J), although changes in tidal volume (V_T_) and respiratory rate (RR) did not reach statistical significance. The effect was particularly pronounced during inspiratory flow-limited breathing in REM sleep with J60 significantly increasing maximal inspiratory flow (V_I_max) and V_E_ (Figure 1J), which suggests that upper airway obstruction decreased. Notably, both V_T_ and RR were elevated (Figure S4). Activation of LEPR^b^+ neurons in the DMH decreased apnea and oxygen desaturation indices in NREM and REM sleep (Figures 1K, 1L, and S5). Relationships between the number of DREADD-transfected LEPR^b^+ cells and the magnitude of respiratory responses to J60 were examined in five mice, and no correlation was found (p > 0.05; Figure S6). The activation of LEPR^b^+ DMH neurons with J60 did not affect sleep architecture: animals had similar total sleep time, NREM and REM sleep time, NREM and REM sleep bout number, or bout length, compared with saline treatment (Table S1). In contrast, J60 had no effect on respiration in mice transfected with the control virus (Figure S7). Thus, stimulation of LEPR^b^+ neurons in DMH increased ventilation and improved upper airway patency during sleep without affecting sleep architecture.

### Activation of LEPR^b^+ neurons in the DMH increased HCVR and metabolic rate

**Figure 2.**
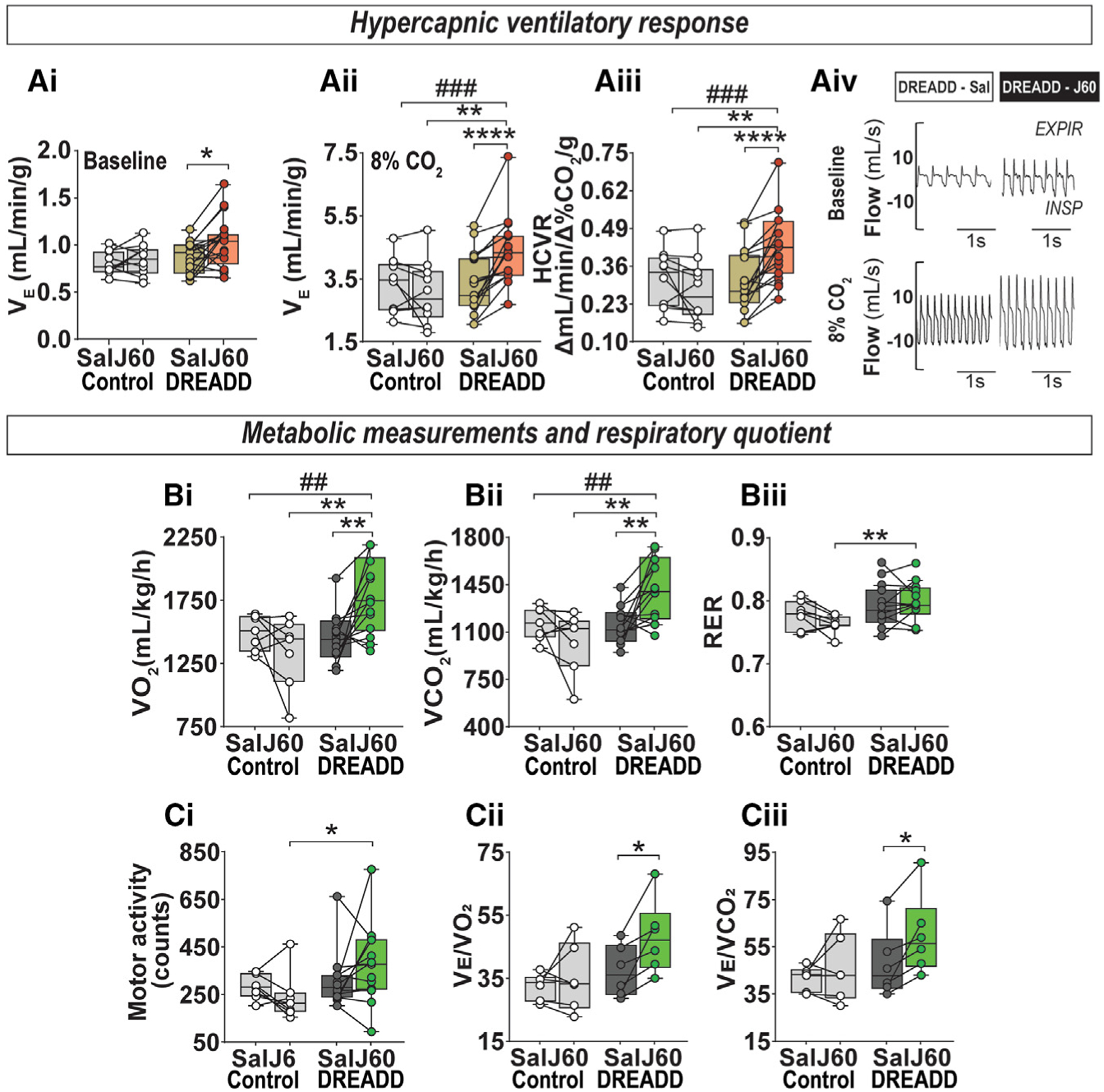
Activation of LEPR^b^ neurons in the dorsomedial hypothalamus (DMH) stimulates breathing out of proportion to increases in metabolism (A) Individual and grouped data show the differences between saline (Sal) and J60 on the baseline minute ventilation (VE) (Ai), VE at 8% inspired CO_2_ (Aii), and hypercapnic ventilatory response (HCVR) (Aiii) in diet-induced obese (DIO) *Lepr*^*b*^-*Cre* mice transfected with control (n = 10) or DREADD (n = 18) virus. Representative traces of HCVR (Aiv). (B and C) Energy expenditure during the light phase in DIO *Lepr*^*b*^-*Cre-GFP* mice transfected with control or DREADD virus. Total oxygen consumption (VO_2_) (Bi), total carbon dioxide production (VCO_2_) (Bii), respiratory exchange ratio (RER) (Biii), total motor activity (Ci), VE/VO_2_ (Cii), and VE/VCO_2_ (Ciii) in the control (n = 6 or 7) and DREADD (n = 6–13) groups. Data are plotted using boxplots (median ± 1.5*interquartile range). ^##^p < 0.01 and ^###^p < 0.001 for the interaction between the virus and J60 effects using repeated-measures two-way ANOVA. *p % 0.05, **p < 0.01, and ****p < 0.001 using Wilcoxonmatched-pairssignedrankorMann-Whitney test.

We tested if activation of LEPR^b^+ signaling in the DMH enhances CO_2_ sensitivity. Hypercapnia rapidly elicited arousals in sleeping animals, so the HCVR was tested only during wakefulness. The ventilatory responses to 8% of CO_2_ challengeare shown inFigure 2A. J60 increased V_E_, both at baseline (room air) and in 8% CO_2_ in DREADD-transfected but not in the control virus-transfected mice. In DREADD-transfected mice, J60 induced a 55% increase in the HCVR compared with saline treatment, and a significant interaction between DREADD and J60 ligand on ventilation and HCVR was observed (Figures 2Aii, 2Aiii, and 2Aiv).

Activation of LEPR^b^+ DMH neurons with J60 elevated VCO_2_, VO_2_, and the respiratory exchange ratio (RER) (Figure 2B). Mouse activity, quantified by the number of infrared beam interruptions, was increased, but the data were highly variable (Figure 2Ci). Increases in the V_E_/VO_2_ and V_E_/VCO_2_ ratios indicated that breathing was stimulated out of proportion to increases in metabolism (Figure 2C).

### Optogenetic activation of LEPR^b^+ neurons in the DMH induced excitatory postsynaptic currents in the neurons of the DR nucleus

**Figure 3.**
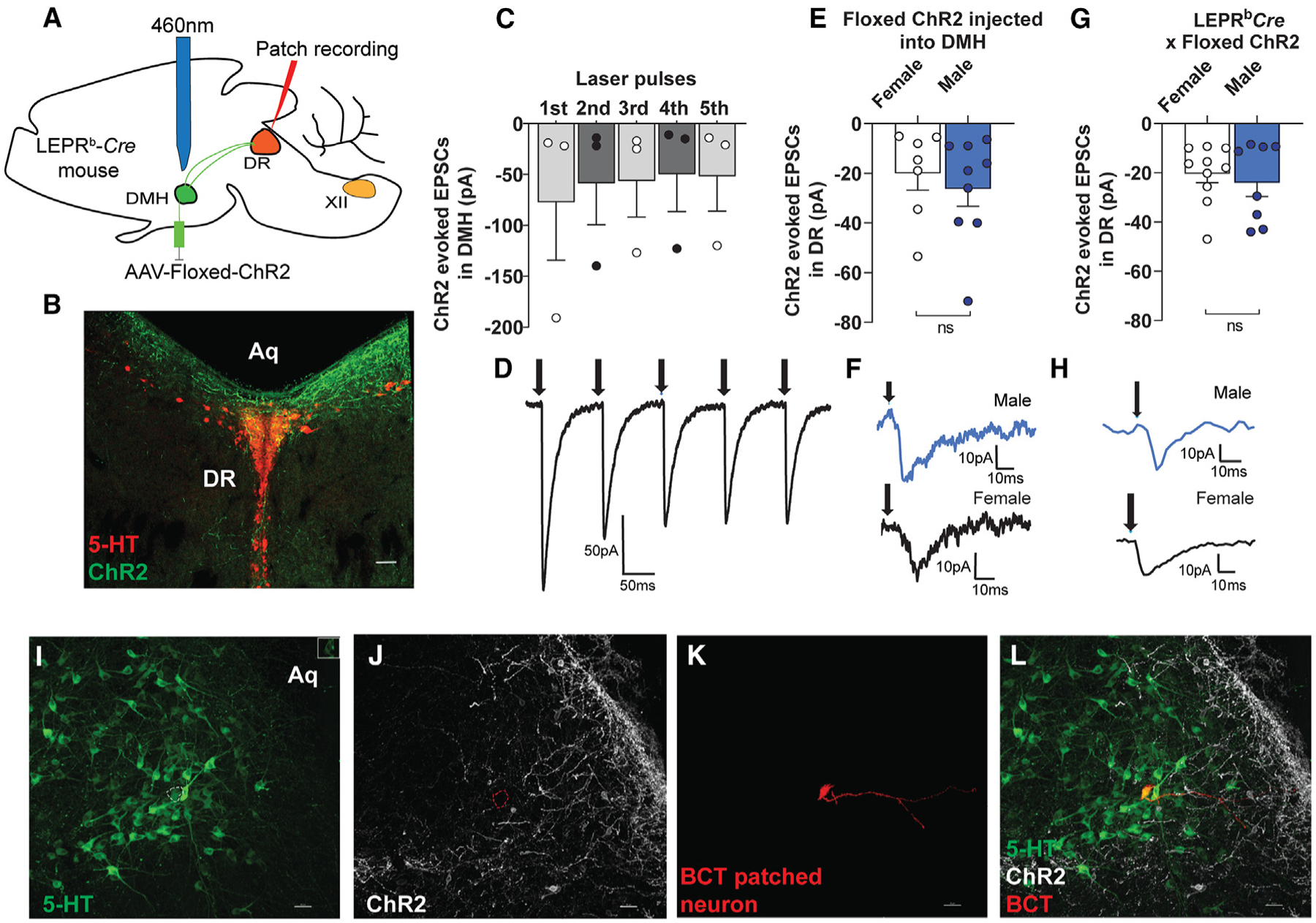
Photostimulation of the LEPR^b^+ DMH neurons evokes excitatory postsynaptic currents (EPSCs) in the dorsal raphe (DR) serotonergic (5-HT) neurons (A) Paradigm of floxed ChR2 injection followed by photostimulation of the DMH and patch-clamp recording of the DR neurons. Sagittal section of the brain containing the DMH ChR2-YFP-labeled LEPR^b^ neurons, fibers, and DR serotonergic (5-HT) neurons. EPSCs and inhibitory postsynaptic currents (IPSCs) were measured in voltage-clamp configuration (B). LEPR^b^+ DMH neurons (green) show projections to the DR nucleus (red 5-HT stain), coronal section; scale bar, 70 μm; (C and D) ChR2 photoactivated responses in LEPR^b^+ DMH neurons (n = 3 from 3 animals). (E and F) Photoactivated EPSCs in 5-HT DR neurons upon activation of LEPR^b^ fibers in *Lepr*^*b*^-*Cre* mice after injection of floxed ChR2-GFP virus into the DMH. (G and H) Photoactivated EPSCs in 5-HT DR neurons upon activation of LEPR^b^ DMH cell bodies in crossbred *Lepr*^*b*^-*Cre*-ChR2 mice. In (F) and (H), top traces from are male and bottom traces from female mice. The values are expressed as means ± SE. Confocal images showing 5-HT-stained neurons in green (I), LEPR^b^ fibers in the DR in white (J), the biocytin (BCT)-filled patch recorded cell in the DR in red (K), and co-localization of the patched 5-HT neuron surrounded by LEPR^b^ fibers (L). Sagittal section; scale bars, 30 μm.

Neural pathways and downstream targets of LEPR^b^+ DMH neurons essential for control of breathing are unknown. To identify projections of DMH LEPR^b^+ neurons, a floxed ChR2 AAV was injected into the DMH of *Lepr*^*b*^-*Cre* mice. The *in vitro* sagittal brain slices contained the DMH ChR2-YFP-labeled LEPR^b^ neurons, fibers, and DR serotonergic (5-HT) neurons. Excitatory postsynaptic currents (EPSCs) and inhibitory postsynaptic currents (IPSCs) were measured *in vitro* in voltage-clamp configuration. As shown in Figures 3 and S8, we detected extensive projections of LEPR^b^+ DMH neurons to 5-HT neurons of DR. EPSCs were examined with patch-clamp recordings from DR neurons *in vitro*. Photoactivation of ChR2 LEPR^b^+ DMH neurons, as well LEPR^b^+ fibers in the DR, evoked robust EPSCs in the 5-HT DR neurons. In contrast to the high density of LEPR^b^+ neurons in the DMH, we found that LEPR^b^+ expressing cell bodies in the DR area are very sparse (Figure S9). Together, these findings indicate that LEPR^b^+ neurons in the DMH send excitatory projections to the serotonergic neurons of the DR, which have been associated with CO_2_ responses.^58,59^

### Effects of LEPR^b^+ DMH neurons stimulation on breathing during sleep and HCVR were blunted by a serotonin receptor antagonist

**Figure 4.**
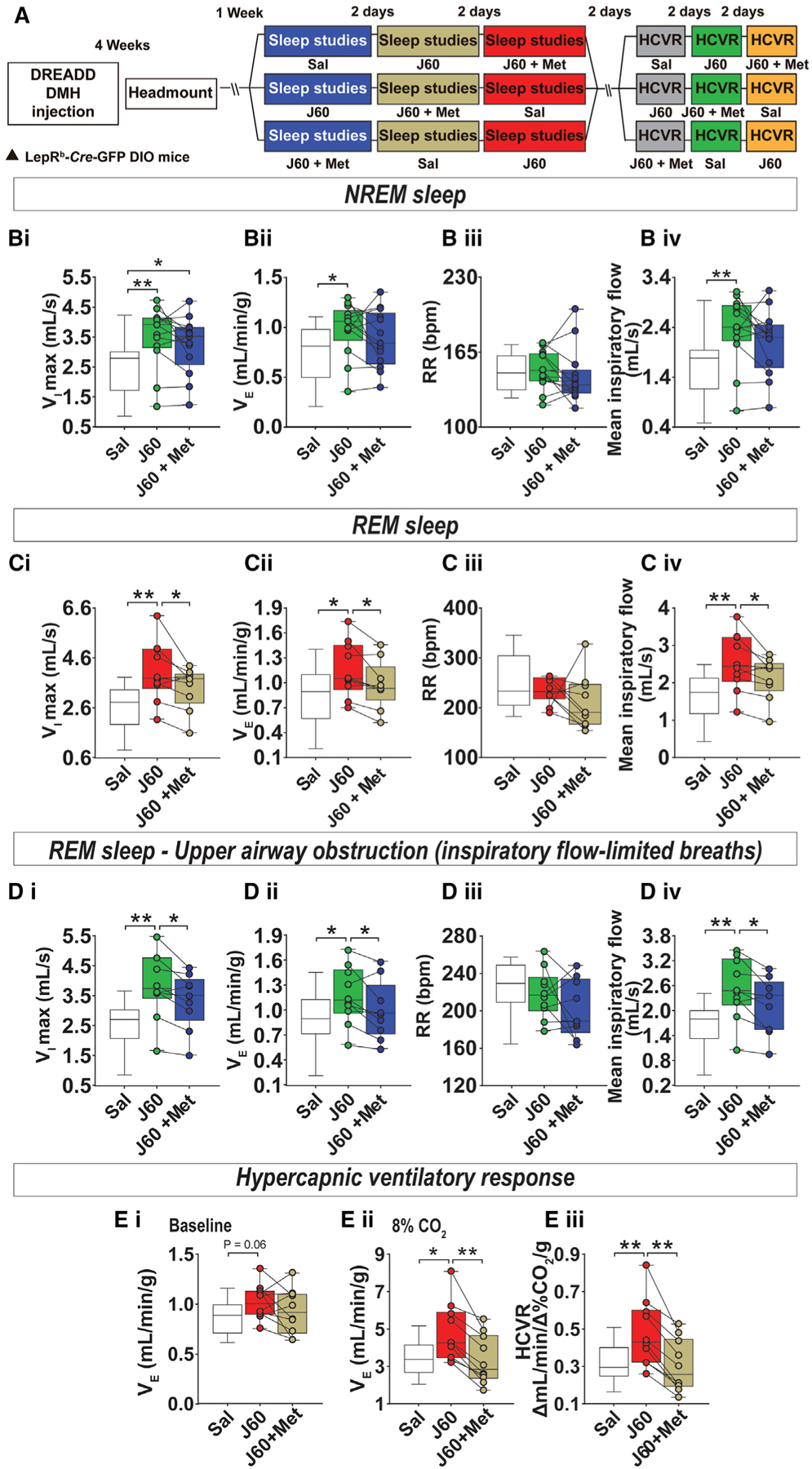
The serotonin receptor antagonist methysergide prevented the increase minute ventilation during sleep and HCVR induced by the activation LEPR^b^ neurons in the DMH (A) Experimental study design. (B–D) Ventilation during sleep in diet-induced obese *Lepr*^*b*^-*Cre* mice transfected with DREADD virus and treated with a serotonin receptor antagonist, methysergide (Met). (B) Individual and grouped data showing the effects of J60 ligand or saline on maximal inspiratory flow (V_I_max) (Bi), minute ventilation (VE) (Bii), respiratory rate (RR) (Biii), and mean inspiratory flow (Biv) during nonflow-limited breathing in NREM sleep in DREADD group mice that received saline (Sal), J60, or J60 + Met. (C) V_I_max (n = 10–13), (Ci), VE (Cii), RR (Ciii), and mean inspiratory flow (Civ) during non-flow-limited breathing in REM sleep. (D) V_I_max, (Di), VE (Dii), RR (Diii), and mean inspiratory flow (Div) during flow-limited breathing in REM sleep. (E) Baseline minute ventilation (VE) (Ei), VE at 8% inspired CO_2_ (Eii) and, HCVR (Eiii) in diet-induced obese (DIO) *Lepr*^*b*^-*Cre* mice transfected with DREADD virus that received Sal, J60, or J60 + Met. Sal group is the same for Figure 1 for comparison purposes. Data are plotted using boxplots (median ± 1.5*interquartile range). *p ≤ 0.05 and **p < 0.01 using Wilcoxon matched-pairs signed rank or Mann-Whitney test.

We performed sleep studies and HCVR measurements in *Lepr*^*b*^-*Cre* DIO mice after the administration of J60 ligand and/or a 5-HT receptor blocker, methysergide^60^ in a crossover, randomized manner (Figure 4A). Methysergide did not significantly attenuate the effects of J60 at baseline (0% CO_2_) breathing during wakefulness (Figure 4Ei) and NREM sleep (Figure 4B), but abolished J60-induced increases in inspiratory flow and V_E_ during both non-obstructed and obstructed breathing in REM sleep (Figures 4C–4D). Methysergide also abolished J60-induced increases in HCVR (Figures 4Eii and 4Eiii). Thus, LEPR^b^+ DMH neurons augment hypercapnic sensitivity and improve upper airway patency during sleep via serotonergic pathways.

### Effects of LEPR^b^+ DMH neurons stimulation on metabolism and breathing were prevented by the elimination of the DR-projecting subpopulation of the DMH LEPR^b^+ neurons

**Figure 5.**
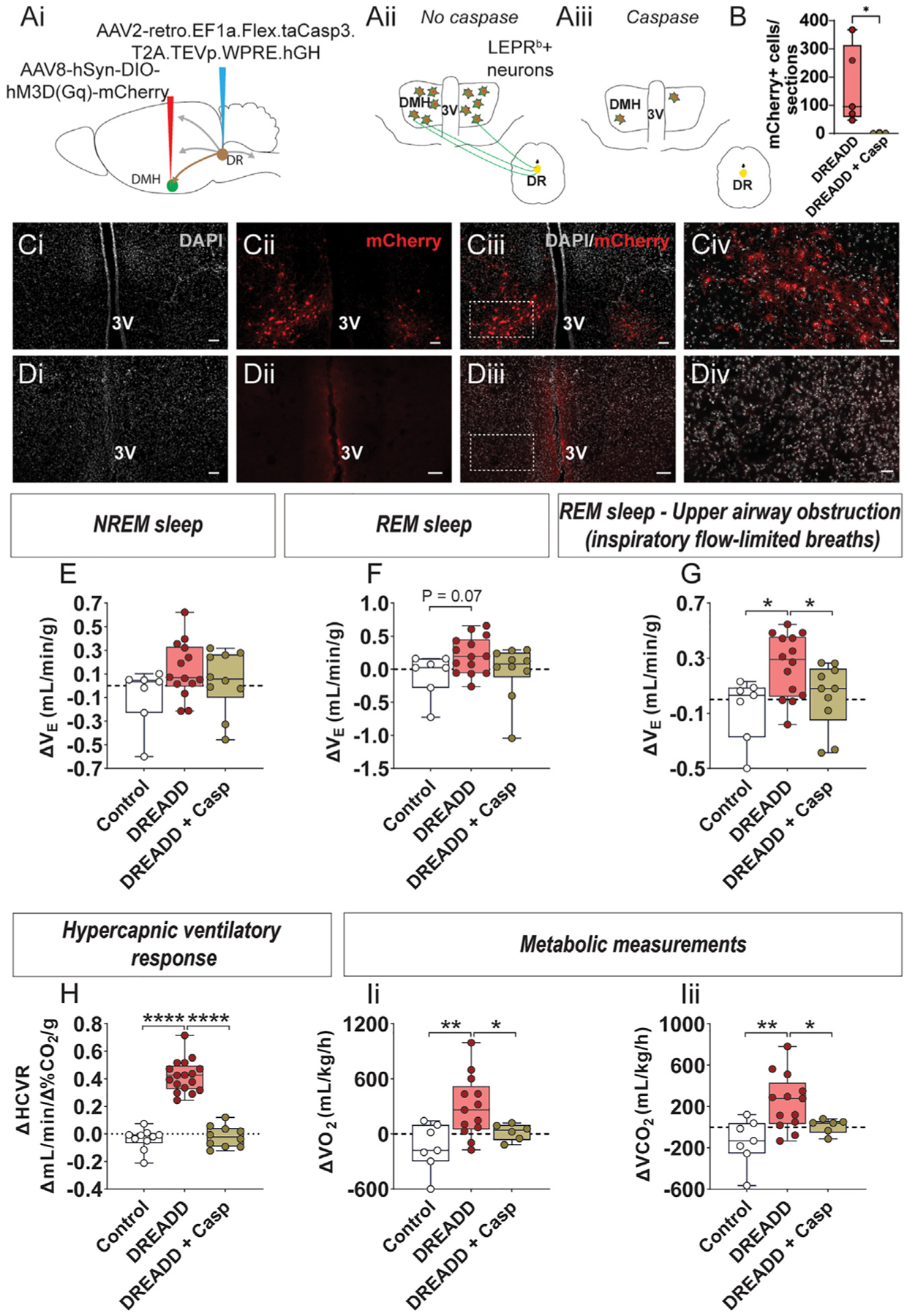
Elimination of LEPR^b^ positive DMH neurons projecting to the DR prevented previously evoked increase in ventilation and metabolism (A) Experimental design, including overall design (Ai). Schematic overview of the hypothesis in *Lepr*^*b*^-*Cre* mice transfected with DREADD into the DMH (Aii) or DREADD into the DMH plus retrograde Cre-dependent AAV harboring caspase to the DR (Aiii). (B) Number of mCherry+ cells per sections (n = 3–5) and (C) histology in the absence of caspase. DAPI represents nuclei (gray, Ci), DREADD in red in *Lepr*^*b-*^*Cre* mice (Cii). Merged image (Ciii). Scale bars, 50 μM. The same at a higher magnification (Civ). Scale bars, 20 μM. (D) Histology in the presence of caspase. DAPI (gray Di), note a significant reduction in DREADD (red) in *Lepr*^*b*^-*Cre* mice that received Cre-dependent AAV harboring caspase to the dorsal raphe (Dii). Merged image (Diii). Scale bars, 50 μM. The same at a higher magnification (Div). Scale bars, 20 μM. 3V, third ventricle. (E–I) Difference (delta) between respiratory and metabolic effects of J60 in *Lepr*^*b*^-*Cre* mice transfected to the DMH with control virus only, DREADD virus only, or DREADD virus plus retrograde Cre-dependent caspase to the DR. Individual and grouped data show the delta between J60 ligand and saline on minute ventilation (V_E_) during non-flow-limited breathing in NREM sleep (E), non-flow-limited breathing in REM sleep (F), flow-limited breathing in REM sleep (G), and hypercapnic ventilatory response (H). (I) Metabolic measurements, total oxygen consumption (VO_2_) (Ii), and total carbon dioxide production (VCO_2_) (Iii) (n = 10). Control and DREADD group are the same for Figures 1 and 2 for comparison purposes. Data are plotted using boxplots (median ± 1.5*interquartile range). *p ≤ 0.05, **p < 0.01, and ****p < 0.0001 using Mann-Whitney test.

We hypothesized that the respiratory and metabolic effects of LEPR^b^+ DMH neurons can be attenuated by the deletion of downstream 5-HT neurons. A retrograde AAV harboring *Cre-*dependent caspase, AAV2-retro.EF1a.Flex.taCasp3.T2A.TEVp. WPRE.hGH, was administered to the DR in *Lepr*^*b*^-*Cre* mice (Figure 5A). Comparison of DREADD DMH deployment without caspase treatment (Figure 5C) and with caspase treatment (Figure 5D) shows that retrograde traffic of caspase from DR to DMH eliminated >99% of mCherry-positive LEPR^b^+ DMH neurons (Figure 5B). Caspase-mediated ablation of LEPR^b^+ DMH neurons abolished J60-induced increases in V_E_ in REM sleep, affecting both non-flow-limited and flow-limited breathing (Figures 5E–5G), HCVR (Figure 5H), VO_2_, and VCO_2_ (Figure 5I). Thus, activation of LEPR^b^+ DMH neurons that project to the DR are necessary and sufficient for the respiratory and metabolic effects of LEPR^b^+ DMH neuron activation. Elimination of the DR-projecting subpopulation of the DMH LEPR^b^+ neurons did not affect sleep architecture (Table S2).

### Elimination of DMH LEPR^b^+ projecting to the DR prevented the increase in ventilation during sleep induced by intranasal leptin

**Figure 6.**
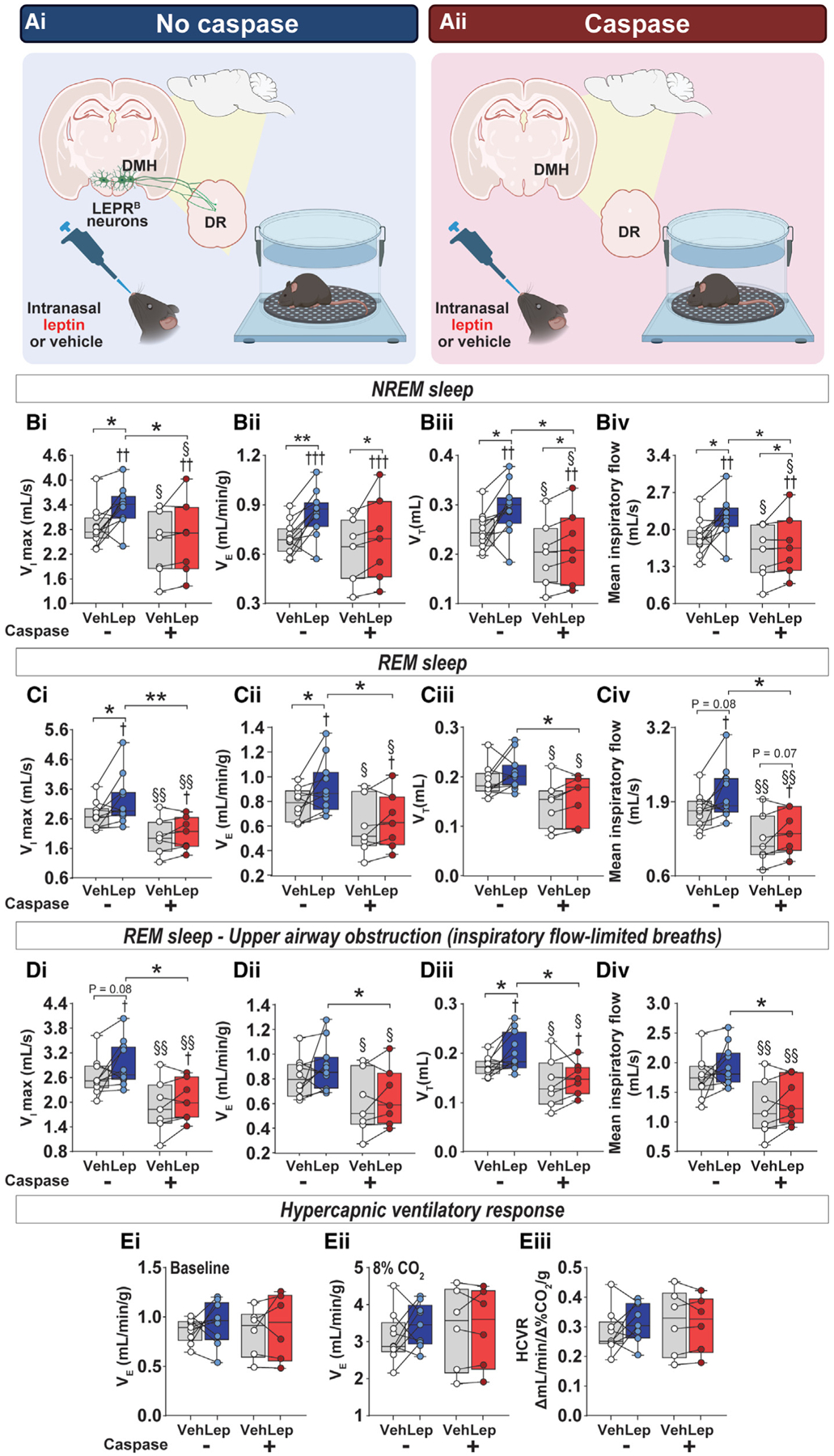
Elimination of LEPR^b^-positive DMH neurons projecting to the DR prevented the increase in ventilation during sleep induced by intranasal leptin (A) Experimental study design showing intranasal leptin or vehicle treatment in diet-induced obese (DIO) mice with intact LEPR^b^-positive DMH neurons projecting to the dorsal raphe (DR) (Ai) (n = 9–11). Effects of intranasal leptin or vehicle in *Lepr*^*b*^-*Cre* DIO mice after caspase-induced elimination of LEPR^b^-positive DMH neurons projecting to the DR (Casp) (Aii) (n = 6 or 7). (B–D) Individual and grouped data show the effects of intranasal leptin or vehicle on maximal inspiratory flow (V_I_max) (Bi), minute ventilation (V_E_) (Bii), tidal volume (V_T_) (Biii), and mean inspiratory flow (Biv) during non-flow-limited breathing in NREM sleep. V_I_max, (Ci), V_E_ (Cii), V_T_ (Ciii), and mean inspiratory flow (Civ) show the effects of intranasal leptin during non-flow-limited breathing in REM sleep. V_I_max (Di), V_E_ (Dii), V_T_ (Diii), and mean inspiratory flow (Div) show the effects of intranasal leptin during flow-limited breathing in REM sleep. (E) Hypercapnic ventilatory response (HCVR) measurements while awake, including baseline V_E_ (Ei), V_E_ at 8% inspired CO_2_ (Eii), and HCVR (Eiii). Data are plotted using boxplots (median ± 1.5*interquartile range). *p ≤ 0.05 and **p < 0.01 using Wilcoxon matched-pairs signed rank or Mann-Whitney test. ^†^p % 0.05, ^††^p < 0.01, and ^†††^p < 0.001, effect of leptin using two-way ANOVA or mixed-effects model. ^§^p % 0.05 and ^§§^p < 0.01, effect of caspase virus using two-way ANOVA or mixed-effects model.

We next examined if the stimulatory effects of intranasal leptin on breathing require downstream 5-HT DR neuronal pathways using retrograde DR injection of *Cre*-dependent caspase in obese *Lepr*^*b*^-*Cre* mice (Figure 6A). In the absence of caspase, intranasal leptin increased inspiratory flow and V_E_ in both non-flow-limited and obstructed flow-limited breathing (Figures 6B–6D). In NREM sleep, ablation of LEPR^b^+ neurons that project to the DR with caspase significantly attenuated, but did not abolish, the respiratory effects of leptin (Figure 6B). In contrast, in REM sleep, ablation of LEPR^b^+ DMH neurons that project to the DR with caspase entirely abolished leptin-induced augmentation of control of breathing and upper airway function (Figures 6C and 6D). During wakefulness, neither intranasal leptin nor caspase had any effect on baseline ventilation and the HCVR. Taken together, caspase experiments show that DR-projecting LEPR^b^+ DMH neurons account for all effects of leptin on ventilatory control and upper airway patency during REM sleep.

### The effect of LEPR^b^+ DMH neurons on breathing in female DIO mice

**Figure 7.**
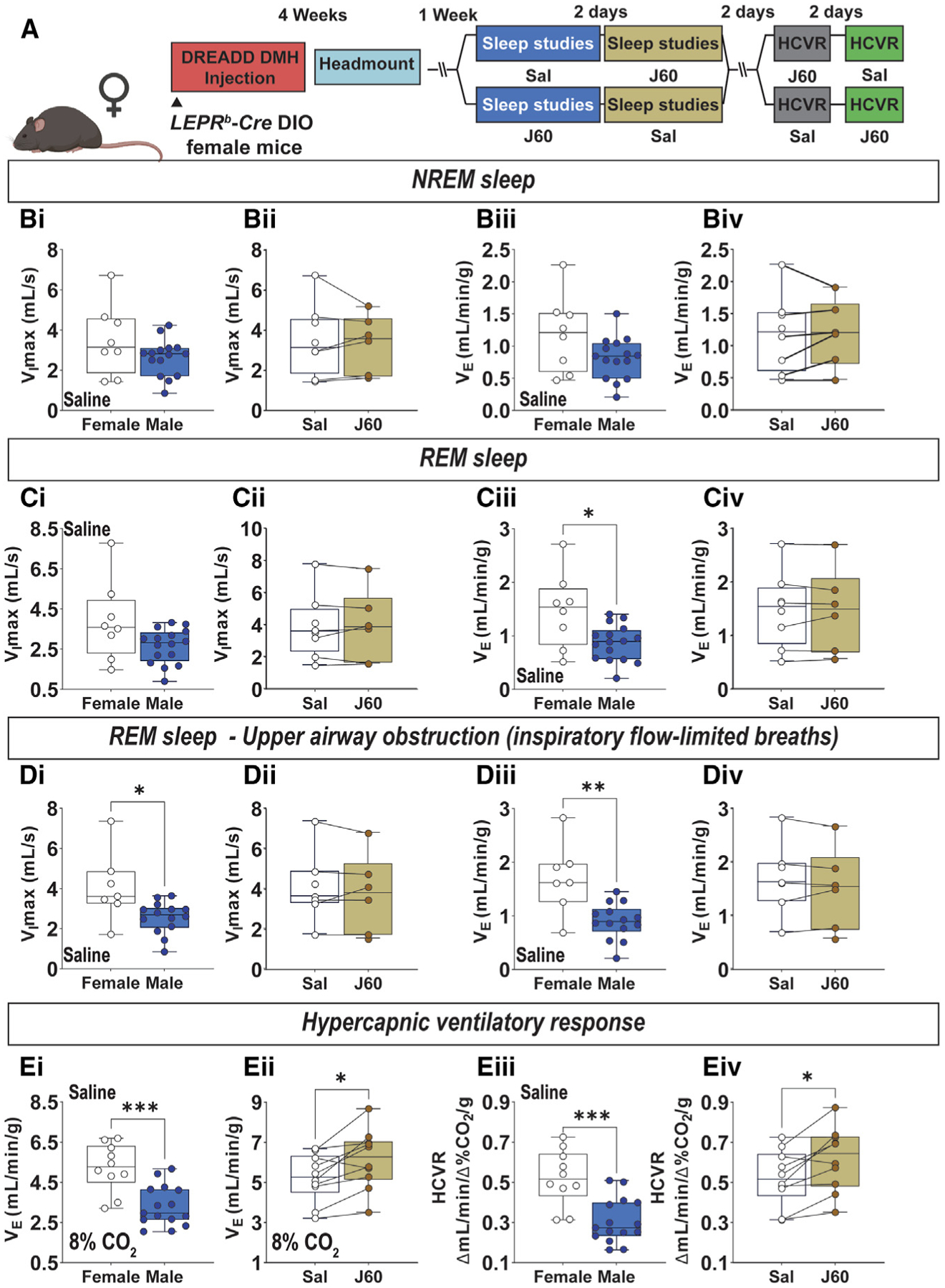
The effect of activation of LEPR^b^ neurons in the dorsomedial hypothalamus (DMH) in Lepr^b^-Cre DIO female mice (A) Experimental study design. (B) Sex differences at baseline (Bi) and the effect of J60 vs. saline (Sal) in females (Bii) on the baseline maximal inspiratory flow (V_I_max) and sex differences at baseline (Biii) and the effect of J60 vs. Sal in females (Biv) on minute ventilation during non-flow-limited breathing in NREM sleep. (C) The same parameters as in (B) during non-flow-limited breathing in REM sleep. (D) The same parameters as in (B) during flow-limited breathing in REM sleep. (E) Sex differences and the effect of J60 vs. saline in females on V_E_ at 8% inspired CO_2_ (Ei and Eii, respectively) and hypercapnic ventilatory response (HCVR) (Eiii and Eiv). n = 8–10. Data are plotted using boxplots (median ± 1.5*interquartile range). *p ≤ 0.05, **p < 0.01, and ***p < 0.001 using Wilcoxon matched-pairs signed rank or Mann-Whitney test.

The data shown above demonstrate that leptin stimulates control of breathing and upper airway function during sleep by acting in the DMH of male mice.^16,47^ As sex is a key factor in respiratory control^61^ we also investigated the involvement of LEPR^b^+ neurons in the DMH in respiratory control in*Lepr*^*b*^-*Cre* DIO female mice (Figure7A). Similar to males, female mice did not have significant upper airway obstruction during NREM sleep, therefore only non-flow-limited breathing was analyzed showing no significant difference between sexes (Figure 7B). Compared with males, female mice showed significantly higher V_E_ during both non-flow-limited and obstructed inspiratory flow-limited breathing in REM sleep (Figures 7C and 7D). During wakefulness, female mice demonstrated a 2-fold higher HCVR compared with males (Figure 7E). Chemogenetic activation of the DMH LEPR^b^+ neurons had no effect on female mice during sleep, but it increased hypercapnic ventilation during wakefulness and augmented the HCVR (Figure 7). Thus, obese female mice exhibit higher indexes of respiratory control and upper airway patency than males which may account for attenuated responses to chemogenetic stimulation of the DMH LEPR^b^+ neurons compared with males.

## DISCUSSION

Hypoventilation is a result of inadequate response to carbon dioxide output. Understanding the neural pathways linking breathing and metabolism is fundamental for future drug development in obesity hypoventilation. Our approach allowed dissecting the effects of leptin pathways on control of breathing and upper airway patency during sleep. The main novel finding of our study is that in DIO male mice, activation of LEPR^b^+ neurons in DMH increased the metabolic rate and hypercapnic sensitivity and augmented breathing during sleep. Furthermore, activation of the specific population of LEPR^b^+-DMH neurons projecting to the serotonergic DR nucleus is necessary and sufficient for these responses. We have also demonstrated that the LEPR^b^+ DMH neurons facilitate control of breathing and upper airway patency acting via the same serotonergic pathway. We have further shown that effects of leptin on upper airway patency and breathing during REM sleep are exclusively mediated by the LEPR^b^+ DMH neurons projecting to DR, whereas other pathways likely contribute to respiratory effects of leptin during NREM sleep. Finally, there were significant sex differences: in response to stimulation of the LEPR^b^+ DMH neurons, female DIO mice did not hyperventilate during sleep, while hypercapnic sensitivity during wakefulness was increased. The lack of response in females could be due to the ceiling effect, as they showed significantly higher ventilation and fewer upper airway obstructions during REM sleep compared with males.

### Leptin acts in the DMH to couple metabolism and control of breathing

We have shown that chemogenetic stimulation of LEPR^b^+ DMH neurons increases VO_2_ and VCO_2_ as well as motor activity and metabolism. These findings are consistent with the previous reports that LEPR^b^+ neurons in the DMH regulate metabolism^45^ by increasing sympathetic tone to the brown adipose tissue even in the presence of leptin resistance^43^ and that LEPR^b^+ neurons in DMH regulate locomotion.^44^ Of note, previous studies reported regional specificity of leptin resistance with some areas of the brain spared, including the DMH.^62–64^ However, these findings were related to the effects of leptin on blood pressure and sympathetic tone regulation, and the relevance of these data for control of breathing has not been determined. Novel findings of our study were that (1) LEPR^b^+ DMH neurons project and stimulate serotonergic neurons of DR, and (2) DR administration of retrograde AAV carrying floxed caspase in a LEPR^b^-dependent manner abolished metabolic effects of LEPR^b^+ DMH neurons. Thus, LEPR^b^+ DMH neurons project to serotonergic neurons of the DR, and the metabolic effects upon activation of the DMH leptin-DR network may occur via their downstream projections to the sympathetic preganglionic neurons.

We demonstrated that stimulation of LEPR^b^+ DMH neurons up-regulated CO_2_ responsiveness during wakefulness and increased maximal and mean inspiratory flow during NREM and REM sleep, suggesting that activation of the HCVR across sleep/wake state. These data confirm our previous report in *db*/*db* mice.^47^ Novel findings of our study were that (1) chemogenetic stimulation was abolished by serotonergic blockade, and (2) retrograde *Cre*-dependent caspase injected into the DR ablated all LEPR^b^+ DMH neurons projecting to the DR and abolished the effects of LEPR^b^+ DMH stimulation on hypercapnic sensitivity in the same manner as it abolished the effects on metabolism. Thus, we can conclude that LEPR^b^+ DMH neurons projecting to DR couple metabolism and breathing.

Intranasal leptin did not increase the awake HCVR, but significantly augmented non-flow-limited breathing during sleep. The effect of intranasal leptin on control of breathing during sleep emulated the effect of LEPR^b^+ DMH neuronal activation. Retrograde AAV *Cre*-caspase administration completely abolished the effect of intranasal leptin on non-flow-limited breathing (i.e., control of breathing) in REM sleep. Taken together with an identical caspase effect on LEPR^b^+ DMH chemogenetic stimulation, our data indicate that the overall effect of leptin on control of breathing during REM sleep is entirely mediated by the LEPR^b^+ DMH neurons projecting to the DR. In contrast, caspase attenuated but did not entirely abolish effects of intranasal leptin in NREM sleep, suggesting that in NREM sleep respiratory effects of leptin are not exclusively mediated by the DMH.

The role of serotonergic pathways in respiratory control is well established, but has mostly focused on medullary serotonergic neurons,^65–67^ rather than the DR. In contrast, serotonergic DR neurons have been implicated in hypercapnia-induced arousals from sleep, but not in the HCVR.^58^ LEPR^b^+ DMH activation or caspase ablation did not affect sleep architecture. The DR neurons likely relay the leptin stimulus to respiratory neurons downstream rather than act as an independent regulator of breathing. Alternatively, LEPR^b^+ DMH neurons projecting to DR may also co-project to other serotonergic neurons, such as medullary raphe, which mediate hypercapnic sensitivity. Overall, our data suggest that LEPR^b^ neurons in DMH projecting to 5-HT neurons of DR integrate VCO_2_ and ventilatory responses to CO_2_.

### Leptin acts in the DMH to improve upper airway patency during sleep

Inspiratory flow limitation is defined by the flattening or plateau in the airflow at the maximal level (V_I_max) in the presence of increased effort.^68–71^ Inspiratory flow limitation is the defining feature of upper airway obstruction during sleep (i.e., OSA).^71–75^ We have previously shown that leptin-resistant DIO mice develop inspiratory flow limitation during sleep, especially during REM sleep,^13^ which was similar to obese humans.^76,77^ Upper airway obstruction has been acutely relieved by intranasal leptin, but not by systemic leptin administration.^34^ Intracerebroventricular leptin administration had a similar effect^16^ and *in vitro* data in brain slices showed that leptin can stimulate hypoglossal motoneurons.^78^ Taken together, our previous data suggest that leptin improves upper airway patency acting on hypoglossal motoneurons, but the mechanisms remained unclear. Furthermore, we have also shown that hypoglossal motoneurons do not express leptin receptors,^34^ suggesting that the effect of leptin is most certainly indirect and polysynaptic.

Our present data confirm evidence of significant inspiratory flow limitation (i.e., upper airway obstruction) during REM sleep in DIO mice, which was relieved by both stimulation of LEPR^b^+ DMH neurons and by intranasal leptin.The effects of both chemogenetic stimulation and leptin were abolished by (1) 5-HT blockade and (2) targeted ablation of the LEPR^b^+ DMH neurons projecting to the serotonergic neurons of the DR. Previous animal studies showed that serotonergic neurons stimulate hypoglossal motoneurons, especially during REM sleep,^79,80^ although medullary raphe rather than DR neurons have been implicated.^81–83^ Our study indicates that the LEPR^b^+ DMH neurons projecting to DR mediate both leptin/LEPR^b^-DMH-induced augmentation of chemoreflex and activation of hypoglossal motoneurons. Hypercapnic chemoreflex is a critical mechanism terminating obstructive apneas.^84–86^ DR neurons may activate hypoglossal motoneurons directly/monosynaptically or indirectly, for instance via CO_2_-sensing neurons of the retrotrapezoid nucleus.^18^ Alternatively, LEPR^b^+ DMH neurons projecting to DR may have axon collaterals and also projectto the medullary raphe to activate hypoglossal motoneurons. Thus, LEPR^b^+ DMH neurons projecting to DR maintain pharyngeal patency during sleep via serotonergic pathways, but future studies are needed to determine precise mechanisms and neuronal populations by which upper airway motoneurons are stimulated.

### Sex differences

Sex differences in hypercapnic sensitivity in DIO have not been adequately studied in literature,^87^ but the relatively low prevalence of SDB in pre-menopausal women is well known.^6^ To our knowledge, this is the first study showing that female obese mice have markedly higher hypercapnic sensitivity and less severe upper airway obstruction compared with males, which emphasizes the relevance of our model to human disease. However, the effect of LEPR^b^-DMH stimulation on control of breathing and upper airway patency was markedly attenuated in female obese mice compared with males. We speculate that the lack of ventilatory responses to chemogenetic stimulation of the LEPR^b^+ DMH neurons in females is due to the ceiling effect. Thus, female DIO mice appear to be able to preserve their ventilation and chemoreflex and protect their upper airway patency during sleep, regardless of leptin signaling.

### Limitations of the study

Our study has several limitations. First, evidence that serotonergic mechanisms play a role in human SDB is uncertain. Studies in normal volunteers showed that selective serotonin reuptake inhibitors (SSRIs) increase genioglossus electromyogram (EMG).^88^ More recent animal studies questioned the magnitude of the effect and relevance of serotonergic pathways,^89^ while clinical trials were disappointing.^90^ However, our study suggests that leptin-mediated serotonergic pathways may be significant in the settings of obesity hypoventilation combined with upper airway obstruction (90% of patients with OHS and 10%–40% of obese OSA patients). Additionally, our results suggest SSIRs would likely be most effective in conjunction with upstream leptin receptor activation. Second, caspase-induced elimination of LEPR^b^+ DMH neurons projecting to the DR could induce neuroinflammation. However, our data show that caspase abolished effects of J60, which were specific for the LEPR^b^ signaling in the DMH rather than affecting baseline values (Figure 5; Table S2). This argues against a non-specific contribution of neuroinflammation. Third, although we clearly demonstrate that LEPR^b^+ DMH neurons projecting to DR regulate both metabolism and breathing, the downstream pathways and the subtype of 5-HT receptor implicated in leptin’s action^91^ are unknown. Fourth, caspase could eliminate LEPR^b^+ neurons projecting to DR from other than DMH areas of the brain (Figure 5Ai). However, caspase blunted only the effects of chemogenetic stimulation of the LEPR^b^+ DMH neurons but did not decrease ventilation or metabolism at baseline. It suggests that LEPR^b^+ neurons in DMH projecting to serotonergic DR neurons are responsible for the majority of leptin’s effect on breathing and metabolism.

### Conclusions

We show that projection of LEPR^b^+ neurons in DMH to serotonergic DR neurons is an essential network for coupling VCO_2_ to CO_2_ respiratory sensitivity. Activation of these neurons is critical in preventing obesity hypoventilation and upper airway obstruction during sleep.

## STAR★METHODS

### RESOURCE AVAILABILITY

#### Lead contact

Further information and requests for resources and reagents should be directed to and will be fulfilled by the lead contact, Vsevolod Y. Polotsky, at vsevolod.polotsky@gwu.edu.

#### Materials availability

This study did not generate new unique reagents.

#### Data and code availability

- All data reported in this paper will be shared by the lead contact upon reasonable request.
- This paper does not report original code.
- Any additional information required to reanalyze the data reported in this paper is available from the lead contact upon request.

### EXPERIMENTAL MODEL AND STUDY PARTICIPANT DETAILS

All experimental protocols were approved by the Johns Hopkins University Animal Use and Care Committee (Protocol # MO19M191) and by the George Washington University Animal Use and Care Committee (Protocols # A2023–009 and # A2023–014), and all animal experiments were conducted in accordance with ACUC guidelines. *Lepr*^*b*^-*Cre-GFP* mice were generated by crossing *LepR*^*b*^-*Cre* (B6.129(Cg)*Lepr*^*tm2(cre)Rck*^/J; The Jackson Laboratory #008320) with GFP-floxed mice (B6; 129-*Gt(ROSA)26Sor*^*tm*2Sho^/J; The Jackson Laboratory #004077). Water and food were available *ad libitum*. Mice were fed with a high fat diet (TD 03584, Teklad WI, 5.4 kcal/g, 35.2% fat, 58.4% kcal from fat) until males reached 40g and females reached 35g and used in our experiments at ~30 weeks of age. Aged-matched DIO mice on the C57BL/6J background were also used (The Jackson Laboratory #380050). Mice were housed at standard environmental conditions (24°C–26°C in the 12-h light/dark cycle, 9 a.m.–9 p.m. lights on), until the experiments started. For the *in-vitro* patch clamp experiments, all animal procedures were approved by the George Washington University Institutional Animal Care and Use Committee.

### METHOD DETAILS

#### Experimental design

1. Protocol #1: To study the effects of activation of LEPR^b^+ DMH neurons in ventilation during sleep of male *LepR*^*b*^-*Cre*-*GFP* diet-induced obese (DIO) mice in a randomized crossover study. We expressed DREADDs or Control virus selectively in the LEPR^b^ + neurons of the DMH. We performed a randomized cross-over study in which mice were treated either with saline i.p. or with the DREADD ligand J60, 0.1 mg/kg i.p, (Hellobio, #HB6261) followed by a sleep study. 2 days later, mice, which received saline, were treated with J60 and vice versa and a sleep study was repeated.
2. Protocol #2: To study the effects of activation of LEPR^b^+ DMH neurons in hypercapnic ventilatory response and metabolism (oxygen consumption, VO_2_, and carbon dioxide production, VCO_2_) in male *LepR*^*b*^-*Cre-GFP* DIO mice in a randomized crossover study as in Protocol #1.
3. Protocol #3: Optogenetic activation of LEPR^b^+ DMH *in vitro. LepR*^*b*^-*Cre* mice were injected into the DMH with AAV expressing photosensitive floxed channelrhodopsin. Four weeks later, mice were sacrificed, sagittal slices of the brain (250 μm) incorporating the hypothalamus, midbrain and brainstem were prepared and excitatory postsynaptic currents (EPSCs) were recorded.
4. Protocol #4: To study the effects of serotonin receptor antagonist in chemogenically-induced stimulation of breathing during sleep and HCVR. DREADDs were expressed in LEPR^b^+ DMH neurons of male DIO mice as in Protocol #1. Sleep studies and HCVR were determined after i.p. saline or J60 +/− a 5-HT receptor blocker methysergide (5 mg kg^−1^, Sigma-Aldrich, M137).^60^ Mice were randomized for the sequential treatment with J60 +/− methysergide, 2 days apart.
5. Protocol #5: Lesion of dorsal raphe-projecting LEPR^b^+ DMH neurons. Male *LepR*^*b*^-*Cre* DIO mice were injected with DREADDs to the DMH as above. We examined examine if the deletion projecting DMH neurons with a retrograde AAV harboring *Cre-*dependent caspase, AAV2-retro.EF1a.Flex.taCasp3.T2A.TEVp.WPRE.hGH administered to the DR in *LepR*^*b*^-*Cre* mice blocks leptin-mediated respiratory stimulation. We performed sleep studies, HCVR, VCO_2_ and VO_2_ measurements as above in mice transfected with the DREADDs only versus DREADDs + retrograde caspase virus.
6. Protocol #6: To study if the stimulatory effects of intranasal leptin on breathing require downstream 5-HT DR neuronal pathways using retrograde DR injection of *Cre*-dependent caspase in male DIO *LepR*^*b*^-*Cre* mice. Intranasal leptin delivery was performed as described previously.^34^ Mice were anesthetized with 1–2% isoflurane. Leptin (0.6 mg kg^−1^, R&D Systems, 498-OB) was prepared in total volume of 24 μL of 1% bovine serum albumin (BSA) in phosphate buffered saline (PBS). Leptin or BSA only (vehicle control) was delivered in 2 doses of 6 μL (3 μL drops) into each nostril. Mice were randomized for the successive treatments with leptin or vehicle in a cross-over-trial as in Protocol #1. We performed sleep studies and HCVR measurements as above in wildtype and *LepR*^*b*^-*Cre* DIO mice versus DREADDs + retrograde caspase virus.
7. Protocol #7: To study the effects of activation of LEPR^b^+ DMH neurons in ventilation during sleep of female *LepR*^*b*^-*Cre* DIO mice. Mice were randomized for the treatment with a single dose of Saline or the DREADD ligand J60 ligand i.p. successively in a cross-over-trial as in Protocol #1 followed by sleep studies and HCVR.

#### Viral vector administration

Viral vector administration were carried out as described previously.^47,57^ In a nutshell, mice were anesthetized with isoflurane for induction (2–3% in the closed chamber) and, after the absence of withdrawal reflex to a firm pinch of tail they were set within the Kopf stereotaxic apparatus (model 963; Kopf Instruments, Tujunga, CA, USA) with mouse adapter VetEquip, and isoflurane vaporizer. Afterward, anesthesia was kept with 1–2% isoflurane. DREADD (AAV8-hSyn-DIO-hM3D(Gq)-mCherry, Addgene, # 44361-AAV8, 4×10^12^ vg.mL^−1^) or Control (AAV8-hSyn-DIO-mCherry, Addgene, #50459-AAV8, 1×10^13^ vg.mL^−1^) virus was delivered bilaterally using pre-pulled glass micropipettes (Sutter, Novato, California) to the DMH using the following stereotactic coordinates from the animal’s bregma: −1.88 mm caudal, ±0.40 mm lateral, and −5.00 mm ventral. For optogenetic electrophysiology experiments, pAAV-EF1a-double floxed-hChR2(H134R)-EYFP-WPRE-HGHpA (AAV1) (≥7 × 10^12^ vg.mL^−1^, Addgene, #20298-AAV1) was injected into the DMH. In a separate group *LepR*^*b*^-*Cre* DIO mice of animals, we administered a retrograde *Cre*-dependent caspase virus AAV2-retro.EF1a.Flex.taCasp3.T2A.TEVp.WPRE.hGH (7×10^12^ vg.mL^−1^ Penn Vector Core – Addgene, #45580) in the dorsal raphe (DR) using the following stereotactic coordinates from the animal’s bregma: −4.6mm caudal and −3.0 ventral. For this experimental protocol, immediately following DR injection, mice were injected with DREADDs to the DMH as above. Buprenorphine (0.03 mg kg^−1^) at the end of surgery to minimize distress.

#### Sleep studies

Polysomnography was performed in a two-arm crossover study design for sleep studies (Figure 1), in which Sal or J60 ligand treatments were performed successively in the same mice, 2 days apart (Figure S1). For headmount implantation, mice were anesthetized with isoflurane 1–2% and placed in the stereotaxic system (Model 963 with 923-B Head Holder, David Kopf Instruments, Tunjunga, CA) in accordance with previous studies.^47,57^ Headmount (no. 8201, Pinnacle Technology, Lawrence, KS) was implanted for electroencephalogram (EEG) and electromyogram (EMG) recordings. Briefly, 4 EEG electrode screws were placed bilaterally in the frontal and parietal bones and 2 EMG leads were tunneled subcutaneously and placed over the nuchal muscle posterior to the skull. Dental acrylic (Lang Dental, Wheeling, IL) was used to secure the headmount in place. Immediately after the surgeries, all mice received 0.03 mg kg^−1^ of Buprenorphine intraperitoneally and were housed in a recovery chamber under heat. Mice were monitored and received additional Buprenorphine if signs of distress or pain were observed.

We used our modified whole-body plethysmography (WBP, EMMS, Hampshire, UK) chamber system for polysomnography to measure tidal airflow and sleep-wake state continuously, generating high-fidelity tidal volume and airflow signals, as previously described.^13,15,47^ After a one-week recuperation period, mice were acclimated to the chamber. Saline or the DREADD ligand J60^92–94^ was injected IP at 0.1 mg/kg i.p. followed by sleep studies as previously described.^16^ The chamber was calibrated to allow high-fidelity tidal volume and airflow signals. On the day of the experiments, mice were placed in the WBP chamber to be recorded from 10:00a.m. to 4:00p.m. on the day of the study (~6 h). Mouse weight and rectal temperature was measured at the beginning and end of the sleep study. During full polysomnographic recordings, the chamber was humidified and kept at ~29°C while a slow leak allowed atmospheric pressure equilibrium. The WBP’s reference chamber filtered out ambient noise from the pressure signal acquired by a transducer. Positive and negative pressure sources were utilized in series with mass flow controllers (Alicat Scientific) and high-resistance elements to generate a continuous bias airflow through the animal chamber while maintaining a sufficiently high time constant. Drorbaugh and Fenn equation was used to calculated the tidal airflow from the plethysmography chamber pressure signal,^95^ which required the measurements of mouse rectal temperature, chamber temperature, room temperature, relative humidity, and chamber gas constant, calculated by utilizing the chamber pressure deflection of a known volume injection. The tidal volume signal was differentiated electronically to generate an airflow signal.

All signals were digitized at 1,000 Hz (sampling frequency per channel) and recorded in LabChart 7 Pro (Version 7.2, ADInstruments, Dunedin, NZ). Sleep-wake state was scored visually in 5 s epochs based on standard criteria of EEG and EMG frequency content and amplitude, as previously described.^13,14,16,47,57^ Wakefulness was characterized by low-amplitude, high-frequency (~10–20 Hz) EEG waves and high levels of EMG activity compared with the sleep states. NREM sleep was characterized by high-amplitude, low frequency (~2–5 Hz) EEG waves with EMG activity considerably less than during wakefulness. REM sleep was characterized by low-amplitude, mixed frequency (~5–10 Hz) EEG waves with EMG amplitude either below or equal to that during NREM sleep. Respiratory signals were analyzed from all REM sleep periods and from periods of NREM sleep sampled periodically at 20-s stretches every half an hour throughout the total recording time. Custom software was used to demarcate the start and end of inspiration and expiration for subsequent calculations of timing and amplitude parameters for each respiratory cycle.

We utilized each breath’s respiratory characteristic to describe maximal inspiratory airflow (V_I_max) and components of minute ventilation (V_E_). We developed an algorithm using the airflow and respiratory effort signals to determine if a breath was classified as inspiratory airflow limited, defined by an early inspiratory plateau in airflow while effort continued to increase. The software provided peak flow values during the first half (V_I_max1), midpoint (V_I_50), and second half (V_I_max2) of inspiration. Breaths resembling sniffs were initially defined as non-flow limited by their short duration, having an inspiration time with a *Z* score lower than 1.75. Breaths having sufficient inspiration time were then classified as inspiratory flow limited if a mid-inspiratory flow plateau was present.^13,14,16^ All parameters were scored by one investigator, who was blinded to experimental conditions.

#### Apnea scoring

We measured apnea index across sleep stages in accordance with our previous study.^96^ Apneas were scored as ≥ 90% reduction in airflow for a period corresponding to two or more breath cycles or ≥0.7 s based on average respiratory rate at baseline.

#### Oxygen desaturation index (ODI)

The oxygen desaturation index (ODI) was defined as ≥4% oxyhemoglobin desaturation from the baseline for at least two breaths per hour of sleep.^13,34^

#### Hypercapnic ventilatory response (HCVR)

HCVR measurements were performed in accordance with our previous at 30°C in a neonatal incubator (Draeger 8000 IC), which has been adapted for respiratory measurements.^97^ Mice were acclimatized before the measurements. HCVR was measured during the light phase. Mice were exposed to a gas mixture of 8% of CO_2_, 21% of O_2_, and balanced in nitrogen. Mice were acclimated with a continuous bias flow controlled with mass flow controllers at room air for 20 min. For exposure, room air was switch to the hypercapnic mixture, and analyses were done after 1 min of exposure when the ventilation reached a plateau. Tidal volume (V_T_), respiratory rate and minute ventilation (V_E_) were measured in mice at baseline (room air) and HCVR was determined in each animal by the slope of the relationship between minute ventilation (V_E_) and inspired CO_2_ (0–8%) during wakefulness via linear least-squares regression analysis.

#### Metabolic measurements

Metabolic studies were performed as previously described.^47,96^ Mice were placed in individual Comprehensive Laboratory Animal Monitoring System (CLAMS) units (Oxymax series; Columbus Instruments, Columbus, OH) for a 24-h acclimation period followed by 24 h of continuous recordings starting at 10:00a.m. The CLAMS units were sealed and equipped with O_2_ electrochemical sensors, CO_2_ infrared sensors and infrared beam movement sensors. Consumed O_2_ (VO_2_) and produced CO_2_ (VCO_2_) were collected every 11 min and measurements were utilized to calculate the respiratory exchange ratio (RER). Mouse activity was recorded with an array of infrared photo beams that surrounded the metabolic cage. As the mice moved, the motor activity was quantified by the number of infrared beam interruptions. Total horizontal and vertical beam breaks were summed and presented as motor activity. Metabolic cages were kept in a 12 h light/dark cycle (7a.m.–7p.m. lights on) with food and water *ad libitum* and a consistent environmental temperature of 29°C–30°C.

#### Optogenetic electrophysiology experiments

Adult *LepR*^*b*^-*Cre* mice were anesthetized with isoflurane, sacrificed and transcardially perfused with ice-cold NMDG aCSF (in mM) (93 NMDG, 93 HCl, 2.5 KCl, 1.2 NaH_2_PO_4_, 30 NaHCO_3_, 25 glucose, 20 HEPES, 5 Sodium Ascorbate, 2 Thiourea, 3 Sodium pyruvate, 10 MgSO_4_.7H_2_O, 0.5 CaCl_2_.2H_2_O, perfused with 95% O_2_ and 5% CO_2_, pH 7.4). The brain was carefully removed and brainstem slices (250 μm) were prepared by vibratome sectioning. The *in-vitro* sagittal brain slices contain the DMH ChR2-YFP-labeled LEPR^b^ neurons and fibers and DR serotonin neurons. The serotonin dorsal raphe neurons were identified by their unique location where they were ventromedial to the aqueduct and dorsal to the superior cerebellum peduncle. Cut slices were then moved from the bath solution and incubated in the NMDG recovery solution (in mM) NMDG 93, HCl 93, KCl 2.5, NaH_2_PO_4_ 1.2, NaHCO_3_ 25, HEPES 20,D-Glucose 25, MgSO_4_ 10, CaCl_2_ 0.5 bubbled with 95% O_2_/5% CO_2_ at 34°C in a water bath for 10 min. The brains slices were then moved to a recording chamber and perfused with standard aCSF solution contained (in mM) NaCl 125, KCl 3, NaHCO_3_ 25, HEPES 5, D-glucose 5, MgSO_4_ 1, CaCl_2_ 2 and continuously bubbled with 95% O_2_/5% CO_2_ to maintain pH at 7.4 at room temperature (22°C–24°C). Whole cell patch clamp techniques were used to record photoactivated ChR2 evoked postsynaptic events in dorsal raphe neurons. Biocytin (0.5%, a highly photostable far-red (excitation laser 640nm) biocytin fluorophore conjugated dye, CF640R, Biotium, Inc., Fremont, CA, USA) was added in the patch solution to further identify the neurons in the dorsal raphe using immunohistochemistry staining.

The patch electrodes were filled with an intracellular recording solution at pH 7.3 containing (in mM): 135 cesium-methanesulfonate, 10 KCl, 10 HEPES, 1 MgCl_2_, 0.2 EGTA, 4 Mg-ATP, 0.3 GTP, 20 phosphocreatine. Lidocaine N-ethyl bromide (1 mg/mL) was included in the intracellular solution to block postsynaptic sodium currents. Photoactivated synaptic currents were elicited by photoactivation of ChR2 expressed in DMH *LepR*^*b*^-*Cre* fibers with 5 pulses of blue light (at a frequency of 10Hz, 5 msec duration, 3mW optic power) or one continuous 5 s exposure (3mW optic power) from a 473-nm laser (Crystal Laser) via a microscope objective. Synaptic events were detected using Clampfit 10.1 and Minianalysis (Synaptosoft, version 5.6.12).

Excitatory and inhibitory postsynaptic currents (EPSCs and IPSCs) were measured in voltage-clamp configuration. Dorsal raphe neurons were held at −55 mV to isolate glutamatergic synaptic transmission and record EPSCs, or +10 mV to isolate GABAergic synaptic transmission and record spontaneous IPSCs. Tetrodotoxin (TTX, 500 nM) and 4-Aminopyridine (4-AP, 100 μM) were included in the bath aCSF to isolate synaptic responses to monosynaptic neurotransmission. At the end of the electrophysiology experiments AMPA and NMDA glutamate receptors were inhibited by adding 6-cyano-7-nitroquinoxaline-2,3-dione (CNQX, 25 μM; Tocris) and D (−)-2amino-5-phosphopentanoic acid (AP5; 50 μM, Tocris) into ACSF, and GABA(A) receptors were inhibited by gabazine (25μM, Tocris). Focal drug application was performed using a PV830 Pneumatic PicoPump pressure delivery system (WPI, Sarasota, FL) to apply drugs from a patch pipette positioned within 30μm from the patched dorsal raphe neuron.

#### Immunohistochemistry, confocal imaging, and image analysis

Expression of the AAV8-hSyn-DIO-hM3D(Gq)-mCherry viruses was confirmed by positive expression of mCherry in the LEPR^b^ positive neurons in the DMH. For the immunofluorescent analysis of the brain, mice were anesthetized with 1–2% isoflurane and promptly perfused transcardially using ice-cold phosphate-buffered saline (PBS; 0.01 M, pH 7.4) solution followed by 4% paraformaldehyde (PF) in PBS. Brains were removed, kept for 4 h in 4% PF and cryoprotected in 30% sucrose overnight at 4°C. Afterward, tissues were embedded in Tissue-Tek O.C.T. (cat# 4583, Tissue Tek, Torrance, CA, USA), and frozen on dry ice. Coronal sections of the frozen brains were cut into 30 μm slices using a cryostat (HM 560, Thermo Scientific, Walldorf, Germany) and tissue was stored at −20°C before further processing. Slides were kept at room temperature for 5 min and then rehydrated with PBS. Sections were covered with mounting medium with DAPI (4,6-diamidino-2-phenylindole; Vectashield, Vector Labs, Burlingame, CA, USA). Images from the different experimental groups were captured and then examined under a Leica TCS SP8 multi-photon confocal microscope equipped with supercontinuum white laser source and single molecule detection hybrid detectors (SMD HyD, Leica, Wetzlar, Germany). A DFC365FX camera at 2048 by 2048-pixel resolution was used for navigation through the sample. Localization of DREADD in the brainstem was confirmed by visualization of mCherry expression. Quantification of GFP+ and mCherry+ cells was done using Imaris 10 software (Oxford Instruments).

The dorsal raphe serotonin neurons filled intracellularly with biocytin (CF640R) were analyzed in confocal images stacks obtained at low (10x) and high (20×) magnification. Biocytin fluorophore was confined to the soma and dendrites of the recorded and filled cell, with negligible spillover in the extracellular space. Serotonin neurons were excluded from analysis if the whole cell patch-clamp recording was stable for less than 20 min, the cell soma was not clearly intact. At the end of each electrophysiology experiment, the slices were fixed in 4% paraformaldehyde (PBS, pH 7.4) overnight, the tissue was processed for serotonin and channelrhodopsin using the following primary antibodies (overnight incubation at 4°C) respectively: rat anti-serotonin antibody (1:250 dilution; Abcam, ab6336, Cambridge, MA), chicken anti-GFP antibody (1:1500 dilution; Abcam, ab13970, Cambridge, MA). Secondary antibodies were goat anti-rat Alexa Fluor 405 and goat anti checken Alexa Fluor 488 (all 1:200 dilution and 4 h incubation at 22°C–24°C; ab175671 and ab150169, Abcam, Cambridge, MA). The brain slices were then mounted and cover slipped with Prolong anti-fade mounting medium (Invitrogen, Eugene, OR), and imaged with a Leica TCS SP8 multi-photon confocal microscope equipped with supercontinuum white laser source and single molecule detection hybrid detectors (SMD HyD, Leica, Wetzlar, Germany). A DFC365FX camera at 2048 by 2048-pixel resolution was used for navigation through the sample. Final images for analysis were taken at 10x from the entire slice, to position the injected neurons and verify its dorsal raphe serotonin neurons. Then z-stacks were taken with 20x/0.75 oil-immersion objective, at z-step size of 2.4 μm to produce volumes allowing identification of colocalization between the patched cells with biocytin labeling and the serotonin neurons. Biocytin injected cells were captured in photon counting regime, an approach allowing for high-dynamic range images to be collected. This was necessary since the injected cell body and the fine distal dendrites represented dramatically different fluorescence signal, which cannot be captured without saturation or missing signal, using more conventional analogue photomultipliers. Images were saved in the “Lif” format, then processed and analyzed using Imaris software (Bitplane Inc. Concord, MA, USA).

Phenotypic identification of the LEPR^b^+ cells in the DMH of the DREADDs treated *LepR*^*b*^-*Cre* mice was assessed by immunostaining for mouse anti-NeuN as a neuronal marker (Millipore Sigma/ MAB377,1:250), rat anti-Glial Fibrillary Acidic Protein (GFAP) as an astrocyte marker, (Invitrogen/13–0300, 1:500), and rabbit anti-ionized calciumbinding adaptor molecule 1 (Iba1) as a microglia marker (FUJIFILM Wako Pure Chemical Corporation/ 019-19741,1:500). Then, the slides were incubated with (all 1:200): Alexa Fluor^™^ 405 Goat anti-Mouse IgG (Invitrogen # A-31553), Alexa Fluorâ 488 Goat Anti-Rat IgG (Abcam, # ab150165), and Alexa Fluor^™^ 647 Goat anti-Rabbit IgG (Invitrogen # A-21245), respectively.

### QUANTIFICATION AND STATISTICAL ANALYSIS

Data were analyzed using Prism, version 9.5.1 (GraphPad Software Inc., San Diego, CA, USA). Mixed-effects model or two-way ANOVA we used to examine whether the factors virus [(DREADD or Control) (DREADD or Caspase)] or drug [(Saline or J60) (Vehicle or leptin) affect the results and whether there is interaction overall. Wilcoxon matched-pairs signed was used to compare differences within groups. Mann Whitney test was used to compare differences between groups. Statistical significance was considered at a level of *p* ≤ 0.05. Descriptive statistics were obtained from the summary tables of each statistical analysis and mentioned in the text as means ± standard error. Graphically, data were plotted using boxplots [median ±1.5*interquartile range (IQR)] showing individual mice.

## Supplementary Material

1

## Figures and Tables

**Table:** KEY RESOURCES TABLE

REAGENT or RESOURCE	SOURCE	IDENTIFIER
Antibodies		
Rat anti-serotonin antibody	Abcam	Cat. # ab6336; RRID:AB_449517
Chicken anti-GFP antibody	Abcam	Cat. # ab13970; RRID:AB_300798
Goat anti-rat Alexa Fluor 405	Abcam	Cat. # ab175671; RRID:AB_2890626
Goat anti checken Alexa Fluor 488	Abcam	Cat. # ab150169; RRID:AB_2636803
Mouse anti-NeuN	Millipore Sigma	Cat. # MAB377; RRID:AB_2298772
Rat anti-GFAP	Invitrogen	Cat. # 13–0300; RRID:AB_86543
Rabbit anti-IBA1	FUJIFILM Wako Pure Chemical Corporation	Cat. # 019–19741; RRID:AB_839504
Goat anti-mouse Alexa Fluor 405	Invitrogen	Cat. # A-31553; RRID:AB_221604
Goat anti-Rat Alexa Fluor 488	Abcam	Cat. # ab150165; RRID:AB_2650997
Goat anti-Rabbit Alexa Fluor 647	Invitrogen	Cat. # A-21245; RRID:AB_141775
Bacterial and virus strains		
AAV8-hSyn-DIO-hM3D(Gq)-mCherry	Addgene	Cat. # 44361-AAV8
AAV8-hSyn-DIO-mCherry	Addgene	Cat. # 50459-AAV8
pAAV-EF1a-double floxed-hChR2(H134R)-EYFP-WPRE-HGHpA (AAV1)	Addgene	Cat. # #20298-AAV1
AAV2-retro.EF1a.Flex.taCasp3.T2A.TEVp.WPRE.hGH	Penn Vector Core/Addgene	Cat. # 45580
Chemicals, peptides, and recombinant proteins		
Methysergide maleate salt	Sigma-Aldrich	Cat. #M137
JHU37160 dihydrochloride (DREADD ligand) - J60 dihydrochloride	Hellobio	Cat. # HB6261
Recombinant Mouse Leptin Protein	R&D Systems	Cat. # 498-OB
Experimental models: Organisms/strains		
LepRb-Cre	The Jackson Laboratory	Cat. #. 008320
ROSA26-EGFP^f^	The Jackson Laboratory	Cat. #. 004077
C57BL/6J DIO	The Jackson Laboratory	Cat. #. 380050
Software and algorithms		
LabChart 7 Pro	ADInstruments	https://www.adinstruments.com/
Clampfit 10.1	Synaptosoft	Version 5.6.12
Minianalysis	Synaptosoft	Version 5.6.12
Imaris	Oxford Instruments	Version 10
Prism	GraphPad Software	Version 9.5.1
BioRender.com		https://www.biorender.com/

## Highlights

- Leptin stimulates breathing and improves upper airway patency during sleep acting in DMH
- DMH LEPR^b^+ neurons send excitatory projections to serotonergic neurons of dorsal raphe
- Leptin regulates breathing and metabolism in obesity through serotonergic pathways
- DMH LEPR^b^+ neurons projecting to the dorsal raphe regulate breathing and metabolism
