# Supplementary material for: Leptin signaling in the dorsomedial hypothalamus couples breathing and metabolism in obesity
Source: Cell Rep. Author manuscript; Available in PMC 2024 Jan 23. (PMC10804286; doi:10.1016/j.celrep.2023.113512)
Supplement: 1 [file NIHMS1954779-supplement-1.pdf]

Cell Reports, Volume *42*

Supplemental information

Leptin signaling in the dorsomedial hypothalamus couples breathing and metabolism in obesity

Mateus R. Amorim, Xin Wang, O. Aung, Shannon Bevans-Fonti, Frederick Anokye-Danso, Caitlin Ribeiro, Joan Escobar, Carla Freire, Huy Pho, Olga Dergacheva, Luiz G.S. Branco, Rexford S. Ahima, David Mendelowitz, and Vsevolod Y. Polotsky

Supplementary material

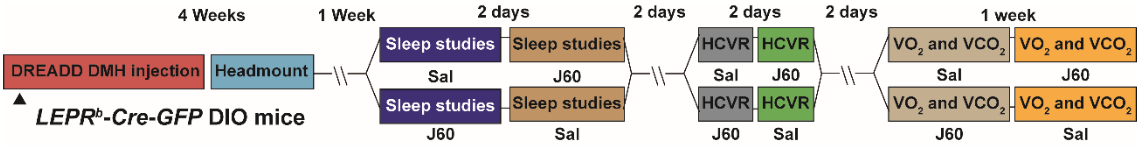

Figure S1: Experimental study design. DREADD, designer receptor exclusively activated by designer drug; DMH, dorsomedial hypothalamus; Sal, saline; VO_2 and VCO_2 , O_2 consumption and CO_2 production measurements.

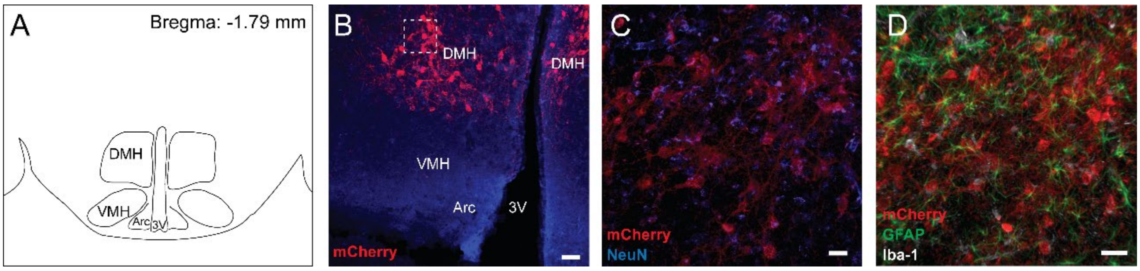

Figure S2: AAV8-hSyn-DIO-hM3D(Gq)-mCherry selectively transfected in the LEPR^b+ DMH neurons. Location of dorsomedial hypothalamus (DMH). arcuate nucleus (Arc), ventromedial hypothalamus (VMH), third ventricle (3V) (A). Low resolution image showing mCherry selectively expressed in the DMH region, scale bar 50µm (B). High resolution image taken from box area in B, showing mCherry labeled LEPR^b+ cells merged with the neuronal marker, NeuN (blue), scale bar 20 µm (C). High resolution image showing that mCherry LEPR^b+ cells do not overlap with astrocytes (glial fibrillary acidic protein, GFAP, green) or microglia (Iba-1, white), scale bar 30 µm (D).

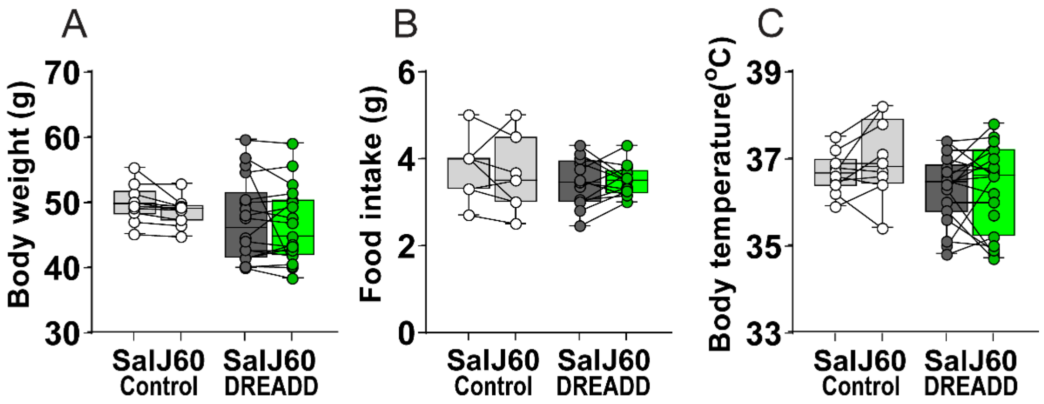

Figure S3: Activation of LEPR^{b} neurons in the dorsomedial hypothalamus (DMH) did not affect body weight, food intake and body temperature. Body weight (A), food intake (B) and body temperature (C).

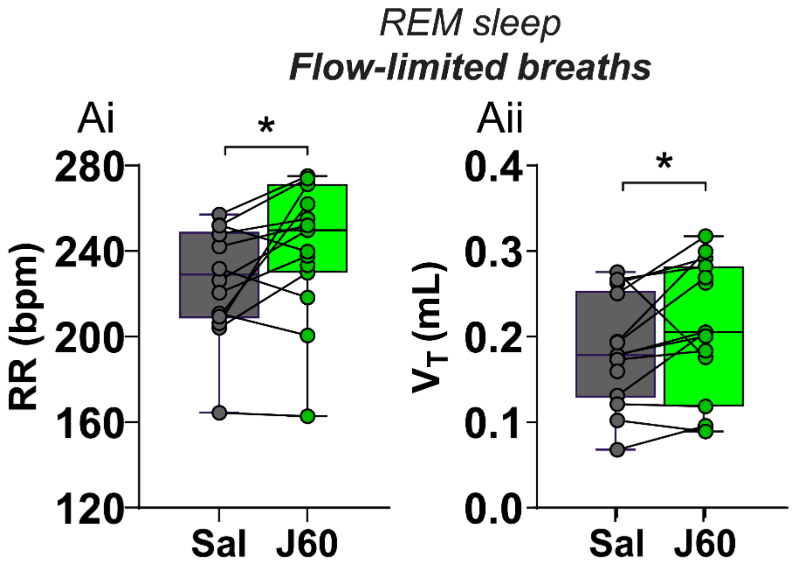

Figure S4: Ventilation during sleep in mice during activation of LEPR^b neurons in the dorsomedial hypothalamus (DMH). Respiratory rate (RR) and tidal volume (V_T) during REM sleep (Ai and Aii) in diet-induced obese *Lepr^b-Cre-GFP* mice transfected with DREADD virus to the DMH (n = 16 – 17). * $p \leq 0.05$, using Wilcoxon matched-pairs signed rank**.**

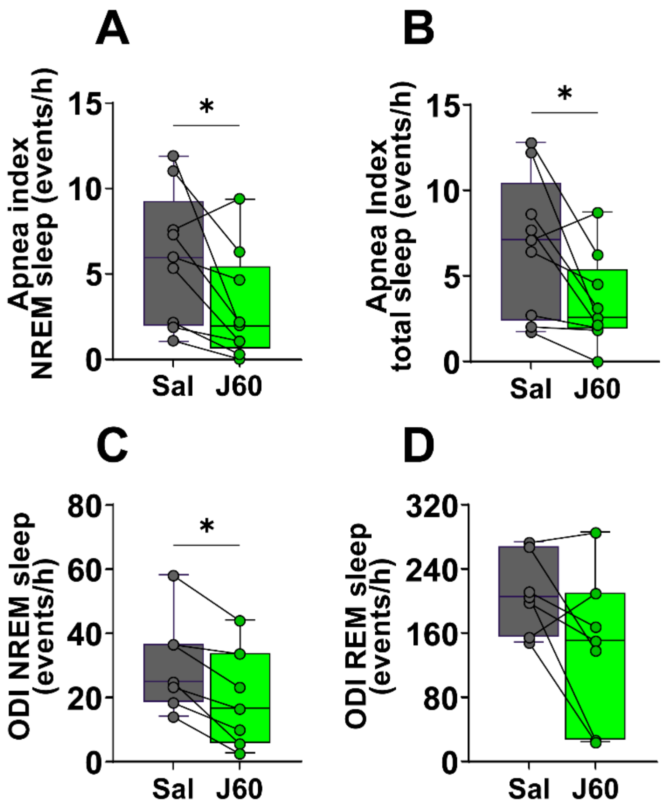

Figure S5: Activation of LEPR[b] neurons in the dorsomedial hypothalamus (DMH) reduced apnea index and oxyhemoglobin desaturation index (ODI). Apnea frequency during NREM sleep (A) and total sleep (B). ODI was defined as a number of oxyhemoglobin desaturations ≥4% from baseline per hour of NREM sleep (C) and total sleep (D). * $p \leq 0.05$, using Wilcoxon matched-pairs signed rank**.**

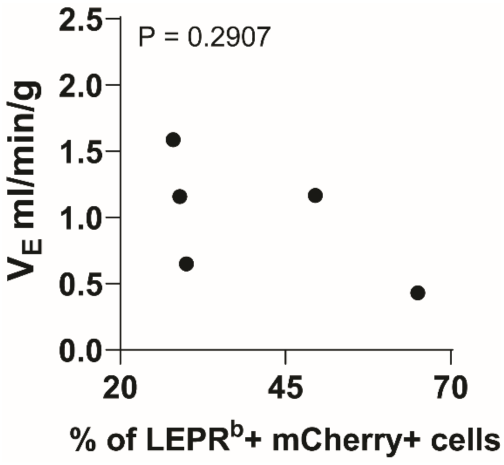

Figure S6: The lack of correlation between percentage of % of LEPR^b+ mCherry+ cells and minute veltination (V_E) in NREM sleep. Pearson correlation test was used for statistical comparisons.

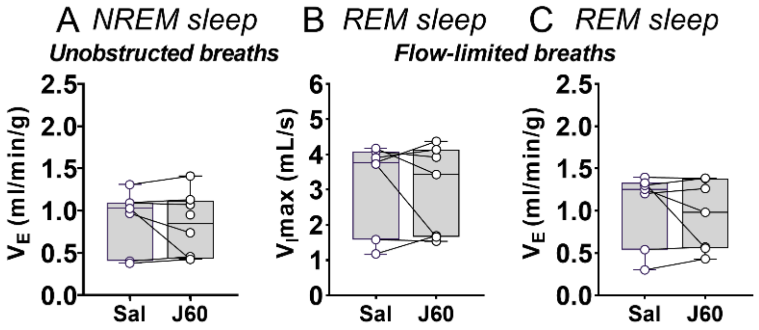

Figure S7: Ventilation during sleep in mice treated with the control virus. Minute ventilation (V_E) during NREM sleep (left, A). Maximal inspiratory flow (V_{imax}, center, B) and V_E during REM sleep (right, C) in diet-induced obese *Lepr^b^*-*Cre* mice transfected with Control (n = 7 – 8) virus**.**

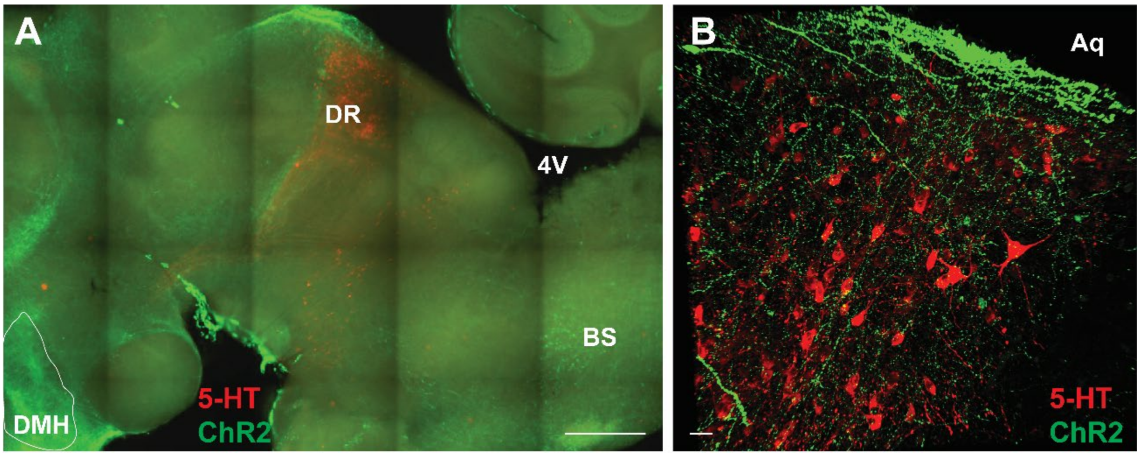

Figure S8: (A). An low magnification confocal image representing ChR2-eYFP labeled DMH and IHC staining DR serotonin (5-HT) neurons in a sagittal brain section, scale bar 500µm. (B) Representative high-resolution confocal image showing ChR2-eYFP labeled fibers (green) surrounding 5-HT neurons (in red), coronal section, scale bar 30µm DMH: dorsomedial hypothalamus; DR: dorsal raphe; Aq: aqueduct; 4V: 4th ventricle; BS: brainstem.

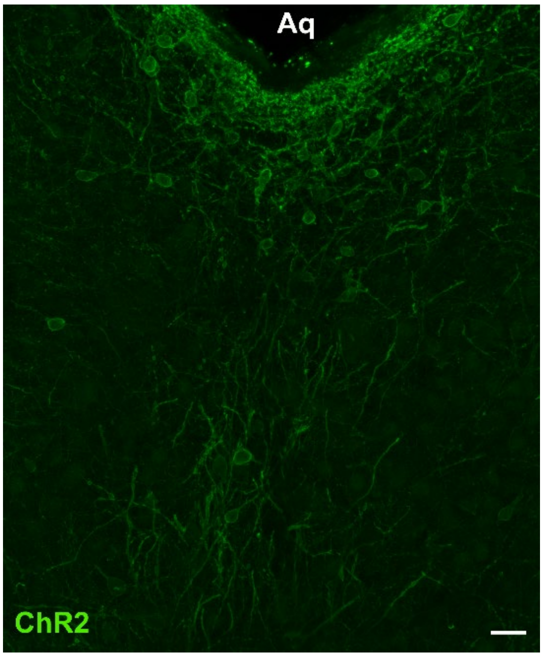

Figure S9: LEPR^{b} fibers and neurons labeled with ChR2 (green) in the dorsal raphe Aq: aqueduct. Scale bars represent 25μm.

Table S1: Sleep architecture and flow limited breaths of diet-induced obese *Lepr^b-Cre* mice transfected with Control or floxed DREADD virus to the DMH.

	Sleep Efficiency (%)	Sleep (min)			Bouts				Flow limited breaths (%)	
					Number		Average Length (min)			
		Total	NREM	REM	NREM	REM	NREM	REM	NREM	REM
Control Saline	61,4 ± 1,6	218,9 ± 7,3	203,6 ± 6,6	15,4 ± 2,2	62,5 ± 3,9	11,6 ± 1,7	3,4 ± 0,3	1,4 ± 0,1	13 ± 7	51 ± 5
Control J60	62,3 ± 2,7	224,6 ± 14,29	209,1 ± 15,1	15,6 ± 2,6	66,5 ± 6,0	12,1 ± 1,9	3,3 ± 0,3	1,2 ± 0,1	21 ± 7	52 ± 7
DREADDs Saline	54,2 ± 1,8 *	176,4 ± 9,67 *	163,5 ± 9,0 *	12,9 ± 1,4	70,7 ± 4,8	11,7 ± 1,3	2,4 ± 0,2 *	1,2 ± 0,1	16 ± 4	45 ± 4
DREADDs J60	56,2 ± 2,9 *	176,4 ± 10,08 *	163,3 ± 8,7 *	13,1 ± 2,4	70,6 ± 5,0	9,6 ± 1,6	2,5 ± 0,2 *	1,4 ± 0,1	18 ± 6	46 ± 5

Values are presented as mean ± SE.
*$p<0.05$

Table S2: Sleep architecture of diet-induced obese *Lepr^b-Cre* mice transfected with DREADD into the DMH plus retrograde Cre-dependent AAV harboring caspase to the dorsal raphe nucleus

	Sleep Efficiency (%)	Sleep (min)			Bouts			
					Number		Average Length (min)	
		Total	NREM	REM	NREM	REM	NREM	REM
DREADDs + Casp (Saline)	61,6 ± 1,8	197,3 ± 10,5	182,0 ± 9,8	15,3 ± 2,3	73,7 ± 3,8	12,1 ± 2,0	2,51 ± 0,2	1,3 ± 0,1
DREADDs + Casp (J60)	59,3 ± 1,5	182,4 ± 8,5	172,2 ± 9,4	10,3 ± 2,0	74,9 ± 6,1	9,1 ± 1,5	2,37 ± 0,1	1,1 ± 0,1

Values are presented as mean ± SE.
*p<0.05
